# Global trends in cardiovascular mortality attributable to high body mass index: 1990–2021 analysis with future projections

**DOI:** 10.1016/j.ajpc.2025.101326

**Published:** 2025-10-07

**Authors:** Parisa Fallahtafti, Hamidreza Soleimani, Shaghayegh Khanmohammadi, Amirhossein Habibzadeh, Morvarid Taebi, Alireza Azarboo, Amirhossein Shirinezhad, Aysan Valinejad, Michael J. Blaha, Sadeer Al-Kindi, Khurram Nasir

**Affiliations:** a Tehran Heart Center, Cardiovascular Diseases Research Institute, Tehran University of Medical Sciences, Tehran, Iran; b Department of Cardiology, Imam Khomeini Hospital Complex, Tehran University of Medical Sciences, Tehran, Iran; c Non-Communicable Diseases Research Center, Endocrinology and Metabolism Population Sciences Institute, Tehran University of Medical Sciences, Tehran, Iran; d School of Medicine, Tehran University of Medical Sciences, Tehran, Iran; e Center for Orthopedic Trans-Disciplinary Applied Research, Tehran University of Medical Sciences, Tehran, Iran; f Johns Hopkins Ciccarone Center for the Prevention of Cardiovascular Disease, Baltimore, MD, USA; g Division of Cardiovascular Prevention and Wellness, DeBakey Heart and Vascular Center, Houston Methodist, Houston, TX, USA

**Keywords:** Body mass index, Cardiovascular disease, Global burden of disease, Mortality, Obesity

## Abstract

**Objective:**

Cardiovascular disease (CVD) remains a major global cause of death, with high body mass index (BMI) as a key modifiable risk factor. This study examines global and regional patterns of CVD mortality attributable to high BMI from 1990 to 2021, with projections to 2032.

**Methods:**

Using Global Burden of Disease 2021 data from 204 countries, we analyzed age-standardized mortality rates (ASMRs) and population-attributable fractions by sex, age, socio-demographic index (SDI), and region. Future trends were estimated using a Bayesian age-period-cohort model, with uncertainity intervals from 1000 posterior simulations.

**Results:**

High BMI-related deaths due to CVD rose from 0.9 million in 1990 to 1.9 million in 2021, with ASMR declining from 24.43 to 22.77 per 100,000 (-6.83 %). High-middle SDI regions had the highest ASMR, while low-middle SDI regions saw the largest increase. Mortality rose for hypertensive heart disease and atrial fibrillation and flutter, but declined for ischemic heart disease and stroke. Older adults accounted for most deaths, though ASMR increased among those aged 15–49. By 2032, deaths are projected to reach 2.5 million (+33 %), with ASMR dropping to 22.06.

**Conclusion:**

Despite modest ASMR declines, high-BMI-related CVD deaths are rising, especially in low-SDI regions, underscoring the need for targeted prevention.

## Graphical abstract

**Figure:**
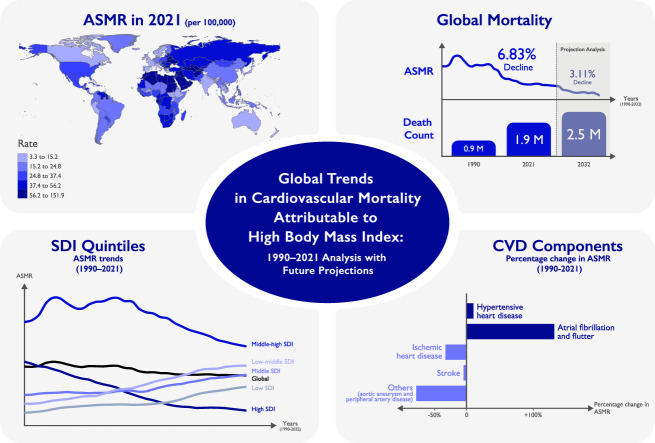

## 1 Introduction

Cardiovascular disease (CVD) remains a leading cause of global mortality. In recent decades, the prevalence of CVD-related conditions has doubled [1]. Between 1990 and 2019, CVD cases surged from 271 million to 523 million, and CVD-related deaths rose from 12.1 million to 18.16 million [1]. Additionally, the global years lived with disability due to CVD increased to 34.4 million in the same period [1]. This upward trend puts an enormous burden on older adults, caregivers, and health systems.

Obesity has emerged as a major global concern, surpassing undernutrition and infectious diseases [2]. Since the 1980s, obesity rates have surged, with nearly one-third of the global population now classified as overweight [3]. Contributing factors to this trend include increased access to high-calorie foods, reduced physical activity, lifestyle changes, sedentary behavior, and genetic predisposition [4]. High body mass index (BMI) is a well-established risk factor for various cardiovascular conditions, including coronary artery disease (CAD), hypertension, heart failure (HF), stroke, and peripheral arterial disease (PAD) [5,6]. Research has shown that obesity contributes to CVD risk through mechanisms such as hypertension, metabolic dysregulation, and reduced cardiorespiratory fitness, leading to higher rates of hospitalization and premature mortality [[7], [8], [9]]. A large meta-analysis of 57 studies found that every 5 kg/m² increase in the BMI raises CVD mortality risk by 40 %, with higher BMI reducing life expectancy by 2–4 years due to CVD [10].

Given the substantial global burden of CVD, understanding its epidemiology is essential for effective public health strategies. Despite preventive and treatment efforts, trends in high BMI-related CVD and intervention effectiveness remain unclear. This study examines global and regional CVD mortality attributable to high BMI from 1990 to 2021, with projections to 2032, offering insights to guide future health policies and interventions.

## 2 Methods

### 2.1 Data sources

This study investigates global and regional trends in CVD mortality linked to high BMI from 1990 to 2021, with forecasts extended to 2032. The analysis utilizes data from the Global Burden of Disease (GBD) 2021 project, a large-scale initiative directed by the Institute for Health Metrics and Evaluation (IHME) at the University of Washington, encompassing 204 countries and territories. The GBD database integrates health data from a wide range of sources, such as national censuses, household health surveys, civil registration and vital statistics, disease-specific registries, healthcare usage records, environmental monitoring systems, and disease notification systems. The GBD 2021 dataset provides estimates for 288 causes of death, 371 diseases and injuries, and 88 risk factors. All data analyzed in this research are publicly available through the GBD Results Tool (https://vizhub.healthdata.org/gbd-results/). Given the diversity of input sources, the accuracy and robustness of the GBD estimates may be influenced by the quality of the underlying data. Detailed descriptions of the data inputs, statistical models, and estimation procedures used in the GBD study have been presented in earlier publications [11,12].

### 2.2 Study outcomes and definitions

The study targets CVD mortality associated with high BMI, as defined by the GBD study, refers to BMI above the Theoretical Minimum Risk Exposure Level (TMREL), which is 20–23 kg/m² for adults aged 20 and older. For children and adolescents aged 2–19 years, high BMI is defined as being overweight or obese based on International Obesity Task Force standards [13,14]. The data were categorized by sex, distinguishing between males and females. Age groups were defined as 15–49, 50–69, and 70+ years, with additional detailed bands (e.g., 50–59, 75–84) to allow for more granular proportion breakdowns. In terms of socio-demographic factors, data were stratified by the socio-demographic index (SDI), which was divided into five quintiles (low, low-middle, middle, high-middle, and high), reflecting income, education, and fertility metrics. Geographic regions were categorized into seven GBD super-regions, which included 1) Central Europe, Eastern Europe, and Central Asia, 2) High-income, 3) Latin America and Caribbean, 4) North Africa and Middle East, 5) South Asia, 6) Southeast Asia, East Asia, and Oceania, and 8) Sub-Saharan Africa along with 204 individual countries and territories. For cause-specific subgroup analysis, we included CVDs for which high BMI was identified as a risk factor, including ischemic heart disease (IHD), stroke, hypertensive heart disease (HHD), atrial fibrillation and flutter (AF/AFL), aortic aneurysm, and lower extremity PAD. It should be noted that, in the GBD classification, HF is considered an impairment rather than a direct cause of death. HF contributes to the burden of many CVDs—excluding aortic aneurysm and PAD—but is not reported as an underlying cause. Instead, its impact is captured under etiologic conditions like IHD and HHD.

### 2.3 Statistical analysis

We analyzed temporal trends in age-standardized mortality rates (ASMRs) and absolute death counts from 1990 to 2021 using the GBD 2021 dataset. ASMRs were calculated using direct standardization with the World Health Organization (WHO) standard population [15]. The contribution of high BMI to CVD mortality was estimated through Population-Attributable Fractions (PAFs). Trends were examined across various subgroups, including sex, age group, SDI quintile, geographic regions, and countries. Cause-specific analyses were also conducted for CVD subtypes. Statistical significance was determined by assessing whether the 95 % Uncertainty Intervals (UIs) of percentage changes excluded zero. All uncertainty estimates were derived from 1000 posterior distribution draws, with the 2.5th and 97.5th percentiles defining the range.

### 2.4 Age-period-cohort analysis

To examine the independent effects of age, period, and birth cohort on high BMI-attributed CVD mortality, an age-period-cohort (APC) analysis was performed. Mortality rates were modeled using a Poisson regression framework with a log-link function [16]. Age was categorized in 5-year intervals, periods spanned 1990–2021 in 5-year increments, and cohorts ranged from pre-1900 to post-2000. The intrinsic estimator method was used to resolve identifiability constraints inherent in APC models. Results were visualized to illustrate age-specific mortality patterns, period-specific risk trends, and cohort-specific risk trajectories. National Cancer Institute’s APC web tool was utilized for analysis and visualization (https://dceg.cancer.gov/tools/analysis/apc) [17].

### 2.5 Future projections

Future trends in high BMI-attributed CVD mortality to 2032 were forecasted using a Bayesian age-period-cohort (BAPC) model. This method combines empirical data with prior distributions for age, period, and cohort effects. This approach applies Gaussian Random Walk (RW) smoothing priors to APC-related trends, preventing excessive deviations between parameter estimates in adjacent time intervals. RW1 assumes temporal consistency by enforcing a constant trend across time intervals, while RW2 accommodates linear temporal changes by assuming a gradual progression. Given the time-varying nature of CVD mortality patterns, the RW2 prior—with its capacity to model linear trends—was selected for predictive analysis to capture evolving mortality dynamics better [18]. Projections were generated using Markov Chain Monte Carlo (MCMC) simulations, yielding median estimates and 95 % UIs for global, regional, and sex-specific outcomes. We performed multiple runs of the Bayesian projection model with alternative prior parameters to evaluate sensitivity. All statistical analyses were conducted using the R software (R for Windows, version 4.1.3, Vienna, Austria) and R Studio version 1.1.463 (Posit PBC, Boston, MA, United States), utilizing packages *BAPC* [19] and *INLA* [20].

## 3 Results

### 3.1 Trends in high BMI-attributed CVD mortality

Between 1990 and 2021, the absolute number of deaths due to high BMI-related CVD rose from 0.9 million to 1.9 million. However, when adjusted for age within populations over time, the ASMR declined from 24.43 per 100,000 persons (95 % UI 13.63–37.3) in 1990 to 22.77 per 100,000 persons (95 % UI 12.87–34.24) in 2021 [total percentage change −6.83 % (95 % UI −14.0 to 1.6); Table 1].

**Table 1:** The mortality burden of cardiovascular disease attributable to high BMI in 1990 and 2021, and its temporal trends from 1990 to 2021.

	Total Death Counts			Age-Standardized Mortality Rates		
	1990, *N* × 1000 (95 % UI)	2021, *N* × 1000 (95 % UI)	Percentage change, %, (95 % CI)	1990, Rate per 100,000 (95 % UI)	2021, Rate per 100,000 (95 % UI)	Percentage change, %, (95 % CI)
Global	863 (477–1316)	1904 (1072–2864)	120.64 (103–141.23)	24.43 (13.63–37.3)	22.77 (12.87–34.24)	−6.83 (−14–1.6)
**SDI Quintiles**						
Low SDI	32 (21–43)	95 (62- 134)	197.85 (139–259.02)	15.6 (10.36–20.98)	20.61 (13.64–29.36)	32.09 (7–58.19)
Low-middle SDI	94 (60–134)	333 (193–489)	252.02 (191–306.93)	17.33 (11.15–24.52)	24.6 (14.77–35.86)	41.96 (16–63.8)
Middle SDI	167 (113–237)	567 (322–845)	238.16 (163–290.48)	19 (12.81–26.79)	22.87 (12.91–34.13)	20.38 (−7–39.5)
High-middle SDI	288 (140–460)	546 (277–863)	89.11 (68–114.85)	32.95 (15.91–52.22)	28.29 (14.36–44.72)	−14.14 (−23– −3.7)
High SDI	277 (132–453)	358 (198–548)	29.03 (15–59.26)	25.37 (12.02–41.3)	16.03 (9.08–24.47)	−36.83 (−42– −24.04)
**Super Regions**						
High-income	278 (132–451)	350 (190–536)	25.74 (7–63.44)	23.42 (11.16–37.87)	14.43 (8.25–21.82)	−38.39 (−45– −23.71)
Central Europe, Eastern Europe, and Central Asia	240 (98–396)	337 (160–545)	40.54 (25–67.39)	54.55 (22.39–90.35)	51.47 (24.55–83.29)	−5.64 (−16–12.07)
Latin America and Caribbean	54 (30–82)	139 (75–215)	156.25 (132–180.14)	26.24 (15.3–39.71)	22.83 (12.51–35.41)	−12.99 (−22– −4.96)
North Africa and Middle East	94 (55–140)	272 (153–400)	187.19 (149–230.46)	63.79 (37.96–94.05)	67.54 (38.92–100.73)	5.88 (−8–21.2)
South Asia	38 (23–56)	210 (115–313)	444.81 (318–584.95)	7.04 (4.25–10.11)	14.79 (8.31–22.09)	110.08 (57–168.02)
Southeast Asia, East Asia, and Oceania	117 (83–157)	471 (265–721)	302.48 (154–430.14)	12.49 (8.25–17.61)	18.33 (10.22–27.79)	46.79 (−9–101.21)
Sub-Saharan Africa	39 (28–52)	123 (81–170)	211.84 (152–272.13)	21.16 (14.71–28.31)	29.76 (19.6–41.84)	40.63 (15–65.86)
**Sex**						
Female	473 (268–724)	991 (566–1486)	109.56 (90–135.48)	24.01 (13.45–36.84)	21.29 (12.11–31.88)	−11.3 (−19– −1.4)
Male	389 (208–598)	912 (478–1393)	134.09 (115–155.24)	24.09 (13.11–36.99)	24.09 (13.2–36.57)	0 (−8–8.5)
**Age groups**						
15–49 years	78 (41–120)	170 (83–262)	117.84 (92–138.56)	2.89 (1.52–4.43)	4.32 (2.11–6.64)	49.54 (32–63.76)
50–69 years	334 (174–522)	672 (342–1030)	100.8 (83–115.77)	49.11 (25.64–76.67)	46.81 (23.84–71.72)	−4.67 (−13–2.44)
≥ 70 years	449 (247–698)	1061 (582–1598)	135.88 (112–167.05)	222.74 (122.54–346.02)	214.69 (117.87–323.44)	−3.61 (−13–9.12)

UI: uncertainty intervals; CI: confidence interval; SDI: socio-demographic index.

**Fig. 1:**
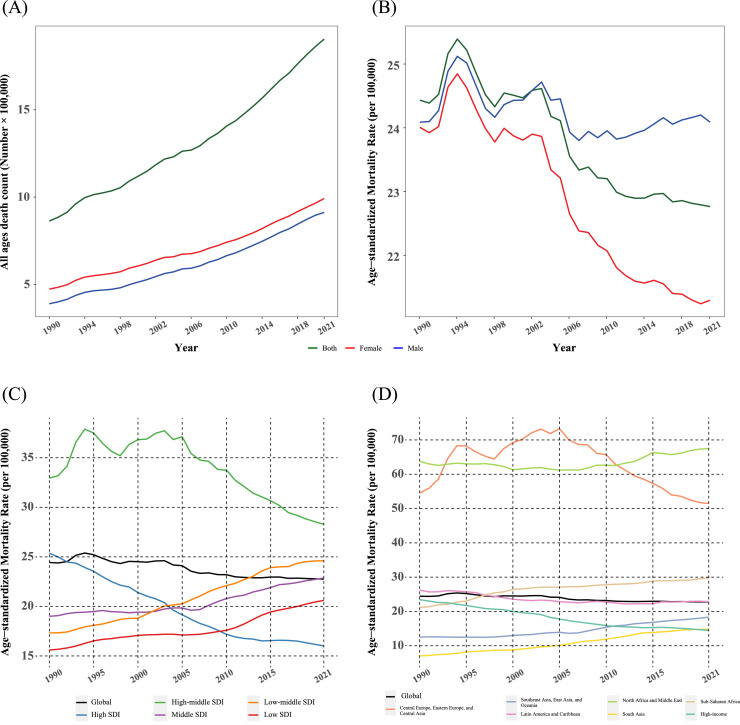
A: Total number of deaths from high BMI-attributed cardiovascular disease (CVD) by sex, 1990–2021. Fig. 1B: Age-standardized mortality rate (ASMR) for high BMI-attributed CVD by sex, 1990–2021. Fig. 1C: ASMR for high BMI-attributed CVD by Socio-Demographic Index (SDI) regions, 1990–2021. Fig. 1D: ASMR for high BMI-attributed CVD by super-region, 1990–2021.

In 2021, the total number of deaths from high BMI-attributed CVD was higher in females (approximately 1 million) than in males (0.9 million; Fig. 1*A*). However, the ASMR was lower in females than in males. The ASMR in females declined from 24.01 per 100,000 (95 % UI 13.45–36.84) in 1990 to 21.29 per 100,000 (95 % UI 12.11–31.88) in 2021 [total percentage change −11.3 (95 % UI −19 to −1.4)]. In contrast, the ASMR in males remained unchanged, at 24.09 per 100,000 in both 1990 and 2021 [total percentage change 0 (95 % UI −8 to 8.5); Fig. 1*B*].

The absolute number of deaths due to high BMI-related CVD increased with age, peaking in individuals aged 70 and older, where deaths rose from 0.4 million in 1990 to 1 million in 2021. ASMR declined in the 50–69 and 70+ age groups but increased among individuals aged 15–49, rising from 2.89 per 100,000 (95 % UI 1.52–4.43) in 1990 to 4.32 per 100,000 (95 % UI 2.11–6.64) in 2021 [total percentage change 49.54 (95 % UI 32 to 63.76)]. The highest proportion of deaths occurred in the 50–59 age group, followed by 75–84, throughout the entire period (*Supplementary Figure 1*).

The mortality from CVD attributed to high BMI varied significantly across SDI regions. In both 1990 and 2021, the highest ASMR was observed in high-middle SDI regions compared to other groups [32.95 per 100,000 (95 % UI 15.91–52.22) in 1990, and 28.29 (95 % UI 14.36–44.72) in 2021]. In 2021, the lowest ASMR was recorded in high SDI regions, at 16.03 per 100,000 (95 % UI 9.08–24.47). In contrast, middle, low-middle, and high-middle SDI regions faced a higher burden of BMI-related CVD as seen in Fig. 1*C*. Between 1990 and 2021, ASMR decreased in high and high-middle SDI regions, while an increasing trend was observed in low, middle, and low-middle SDI regions. The most significant reduction occurred in high SDI regions [total percentage change −36.83 (95 % UI −42 to −24.04)]. Conversely, the most substantial increase was recorded in low-middle SDI regions [total percentage change 41.96 (95 % UI 16 to 63.8)].

**Fig. 2:**
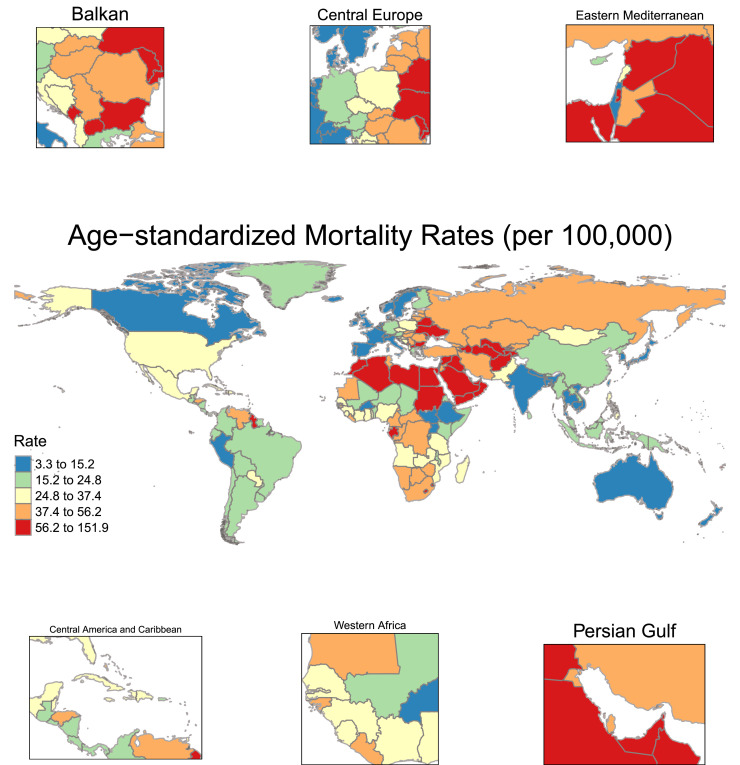
**Global map** showing the age-standardized mortality rate (ASMR) for high BMI-attributed cardiovascular disease (CVD), 2021.

Regional differences in the mortality from high BMI-attributed CVD were evident in 2021. North Africa and Middle East had the highest ASMR at 67.54 per 100,000, followed by Central Europe, Eastern Europe, and Central Asia super-region at 51.47 per 100,000. In contrast, High-income super-region (14.43 per 100,000) and South Asia (14.79 per 100,000) recorded the lowest ASMRs (Fig. 2). Mortality rates significantly declined in High-income [total percentage change −38.39 (95 % UI −45.0 to −23.71)], and Latin America and Caribbean super-regions [total percentage change −12.99 (95 % UI −22.0 to −4.96)]. Despite an overall rise in the number of deaths, the change in ASMRs was not statistically significant in Central Europe, Eastern Europe, and Central Asia, North Africa and Middle East, Southeast Asia, East Asia, and Oceania (*Supplementary Figures 2, 3*). In contrast, South Asia and Sub-Saharan Africa experienced an increasing trend in high BMI-related CVD mortality rates, with South Asia showing the sharpest rise [total percentage change 110.08 (95 % UI 57.0 to 168.02); Fig. 1*D,* 3*A*].

**Fig. 3:**
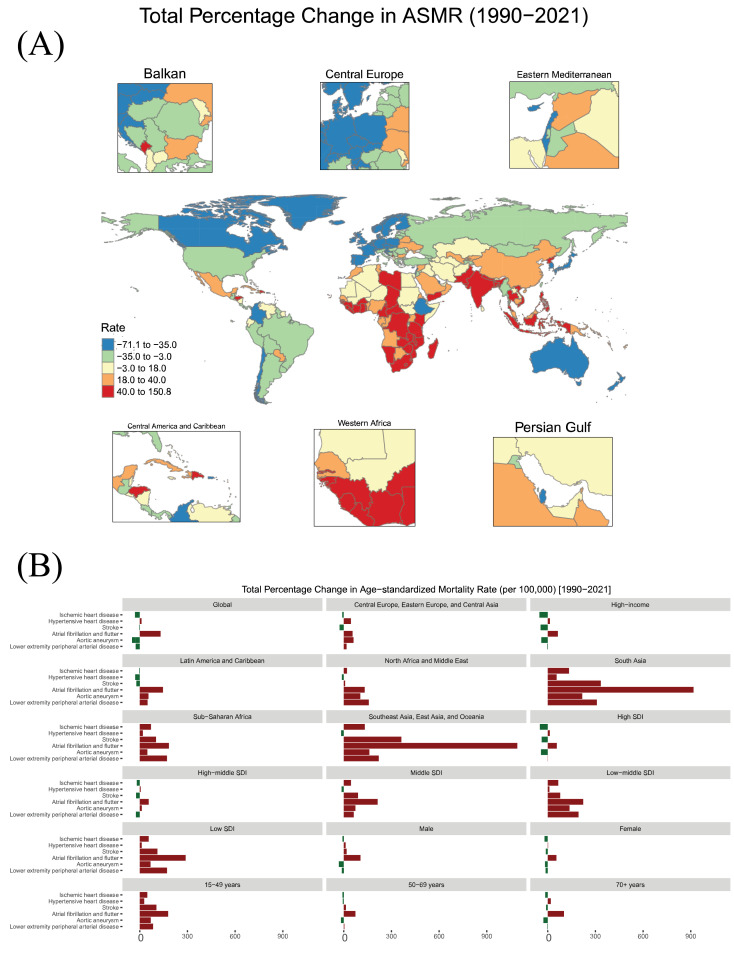
A: Global map illustrating the total percentage change in ASMR for high BMI-related CVD by region, 1990–2021. Fig. 3B: Cause-specific trends in ASMR for high BMI-attributed CVD, 1990–2021.

As expected, the highest absolute number of deaths in 2021 was recorded in China, followed by India, the United States, and the Russian Federation. In terms of ASMR, the highest values were observed in Republic of Nauru [151.95, 95 % UI (67.12–239.36)], Egypt [136.89, 95 % UI (75.82–203.74)], and the Syrian Arab Republic [108.59, 95 % UI (52.88–174.49)], while the lowest ASMRs were reported in Japan [3.28, (95 % UI 1.7–5.11)] and the Republic of Korea [4.58, (95 % UI 2.51–7.38)]. Zimbabwe had the highest increase in ASMR [total percentage change 150.8 (95 % UI 66.0 to 248.89)], followed by Pakistan, Indonesia and India. In contrast, Israel had the most significant reduction in ASMR [percentage change −71.09 (95 % UI −76.0 to −66.01)], followed by Norway, Denmark, and Ireland. The absolute number of deaths and ASMR values for each country are presented in *Supplementary Table 1.*

### 3.2 Cause-specific CVD mortality attributable to high BMI

Analysis of the trends in ASMR for CVD subgroups from 1990 to 2021 revealed distinct patterns across geographical super-regions, SDI, sex, and age groups (Fig. 3*B*). Globally, a downward trend in CVD mortality was observed with aortic aneurysm, IHD, lower extremity PAD, and stroke. This positive trend was particularly pronounced in the High-income super-region, where consistent declines were observed across most analyzed CVD subcategories. However, AF/AFL and HHD showed an increase in ASMR globally. This trend was most pronounced in Southeast Asia, East Asia, and Oceania and South Asia super-regions for AF/AFL, and in South Asia for HHD.

High SDI regions generally mirrored the trends observed in High-income super-region, with consistent decreases in most CVD subtypes’ mortality. In contrast, lower-SDI regions experienced mixed trends, with many CVD types showing increasing ASMR. Stratification by sex revealed similar overall trends in CVD mortality for both males and females, although subtle differences in the magnitude of change were observed across specific CVDs. The decline in IHD and stroke mortality was more prominent in females, while males showed a slight upward trend in some CVD subcategories. Among age groups, the most significant reductions in IHD and stroke mortality occurred in individuals aged 70 and older while, younger age groups (15–49 years) exhibited increasing mortality rates.

### 3.3 Age, period, and cohort effects on high BMI-attributed CVD mortality

**Fig. 4:**
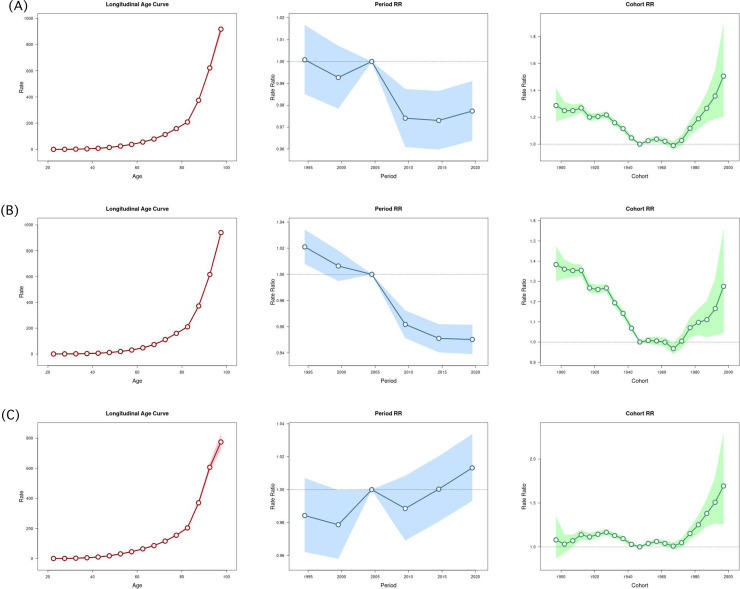
Age, period, and cohort effects on high BMI-attributed CVD mortality, 1990–2021. Panel A presents ASMR for both sexes, Panel B for females, and Panel C for males.

Mortality rates due to high BMI-related CVD increased exponentially with age, with a sharp rise after 60 years. This pattern was observed in both sexes, with the most significant increase occurring in individuals aged 80 and older. Over the study period, mortality risk generally declined, particularly after 2005. However, progress has slowed in recent years, with a slight uptick after 2015. While mortality risk among females continued to decline, males exhibited an upward trend, specifically after 2010. Cohort analysis revealed a declining mortality risk among individuals born after 1900, followed by a sharp reversal between 1960 and 2000, forming a U-shaped trend. Mortality risk steadily declined in cohorts born before 1965 but rose significantly in those born afterward. This shift was more pronounced in females, with both the decline and subsequent rise being steeper, while males exhibited a more gradual change (*Supplemnetary Table 2*). These findings indicate that mortality from high BMI-related CVD rises sharply with age, peaking after 80. Overall risk declined until 2015, though progress has slowed, with males showing rising rates after 2010. Birth-year cohorts reveal a U-shaped trend: declining risk before 1965 and a sharp increase thereafter, especially among females (Fig. 4A–C).

### 3.4 Future projections of high BMI-attributed CVD mortality to 2032

To forecast future trends in high BMI-related CVD mortality, a BAPC analysis was conducted (*Supplementary Tables 3, 4*). By 2032, global deaths due to high BMI-related CVD are expected to rise to 2.5 million, marking a 33 % increase compared to 2021. However, ASMR is projected to decrease to 22.06 per 100,000 in 2032 compared to 22.77 per 100,000 in 2021. The sensitivity analysis shows that overdispersion lowers projections, suggesting more conservative estimates when variability is accounted for. The rate parameter has minimal impact, while higher shape parameters slightly decrease projections (*Supplementary Table 5; Supplementary Figures 4–6*).

In terms of sex, female deaths are projected to reach 1.4 million (ASMR 21.10 per 100,000), while male deaths are estimated at 1.2 million (ASMR 23.46 per 100,000). Despite the declining ASMR, the absolute number of deaths is expected to rise due to population growth and aging. Regionally, ASMR is anticipated to increase in North Africa and Middle East, South Asia, Southeast Asia, East Asia and Oceania, and Sub-Saharan Africa. Conversely, Central Europe, Eastern Europe, Central Asia, Latin America, and High-income super-regions are predicted to experience a decline in ASMR, indicating progress in reducing high BMI-related CVD mortality. Absolute death counts are expected to rise across all world regions, with the most significant increases predicted in South Asia (+427,000 deaths), Southeast Asia, East Asia and Oceania (+312,000 deaths), North Africa and Middle East (+694,000 deaths), and Sub-Saharan Africa (+97,000 deaths).

## 4 Discussion

This study analyzes CVD mortality linked to high BMI from 1990 to 2021 using GBD data. Key findings include: 1) deaths nearly doubled from 0.9 million to 1.9 million, despite a decline in ASMR; 2) high-middle SDI regions had the highest mortality, while low-middle SDI regions showed the fastest ASMR increase; 3) mortality from IHD, stroke, and lower extremity PAD declined globally, but deaths from AF/AFL and HHD increased; 4) females had higher absolute deaths but larger ASMR declines than males, whose rates remained stable; 5) mortality peaked in those 70+, with rising ASMR in younger adults (15–49 years); 6) projections to 2032 indicate rising deaths, especially in South Asia, Southeast Asia, East Asia, and Oceania, though global ASMR may slightly decrease.

In 2021, the highest ASMRs for high-BMI-attributed CVD were observed in North Africa and the Middle East, followed by Central and Eastern Europe and Central Asia, while the lowest rates were found in the High-income super-region and South Asia. Between 1990 and 2021, notable reductions in ASMR occurred in the High-income super-region and Latin America and the Caribbean, reflecting successful public health efforts such as smoking cessation and improved healthcare access [21]. Conversely, South Asia and Sub-Saharan Africa experienced rising mortality, with South Asia’s younger population and lower life expectancy temporarily moderating ASMR despite rapidly increasing obesity prevalence [22]. The elevated ASMR in North Africa and the Middle East is linked to rapid urbanization, dietary shifts, physical inactivity, and metabolic disorders [23].

At the national level, countries like the Republic of Nauru, Egypt, and the Syrian Arab Republic reported the highest ASMRs in 2021, possibly due to factors including weakened healthcare systems, high smoking rates, and unhealthy lifestyles [24,25]. Zimbabwe saw the largest ASMR increase since 1990, likely driven by urbanization and limited public health infrastructure, whereas Israel recorded the largest decline, probably reflecting stronger healthcare and prevention strategies [[26], [27], [28]]. From an SDI perspective, the steepest increases in high BMI-attributable CVD deaths occurred in low and low-middle SDI regions. These areas are undergoing rapid nutrition transitions while lacking adequate healthcare infrastructure and public health capacity [29]. In contrast, high and high-middle SDI regions benefited from more effective prevention, treatment, and health policies—though disparities persist even within these countries, often affecting low-income and marginalized populations [[30], [31], [32]].

Our analysis indicates a significant shift in the global CVD mortality landscape from 1990 to 2021, with a notable increase in ASMR for HHD and AF/AFL attributable to high BMI. While traditional atherosclerotic cardiovascular diseases like IHD and stroke have shown declining trends, the burden of HHD and AF/AFL attributable to high BMI has risen, particularly in regions with low to middle SDI. Improved survival rates for conditions like IHD and stroke, due to better medical treatment, have shifted the mortality burden towards other cardiovascular complications. Moreover, the rise in mortality due to HHD and AF/AFL is likely associated with aging populations. These conditions tend to be more prevalent in older individuals, and as global life expectancy increases, the incidence of HHD and AF/AFL has risen accordingly [33].

Sex-specific analyses revealed a significant decline in ASMR among females but not in males. These findings align with prior research suggesting that females benefit from estrogen-mediated cardiovascular protection, healthier behavioral patterns, and greater healthcare engagement [34]. Conversely, males tend to exhibit higher metabolic risk at equivalent BMI levels and are less likely to adopt weight loss interventions or seek preventive care [35]. Additionally, sex-based differences in fat distribution and cardiometabolic responses contribute to these disparities [36].

Age-stratified results showed that absolute CVD deaths increased with age, peaking among individuals ≥70 years. Despite this, ASMR declined in adults aged 50–69 and ≥70, likely due to better access to care and advancements in secondary prevention [37]. However, individuals aged 15–49 experienced an increase in ASMR, reflecting an alarming rise in early-onset high-BMI-related CVD. This could be driven by increasing childhood and adolescent obesity, as well as early exposure to cardiometabolic risk factors such as hypertension and diabetes [[38], [39], [40]].

Furthermore, a critical finding in our study is the U-shaped pattern observed in the APC analysis, where mortality risk decreased among individuals born before 1965 but increased for those born afterward. This reversal in mortality risk may be associated with significant global lifestyle and dietary changes since the mid-20th century [41]. The global nutrition transition, characterized by adopting Western dietary habits, including increased consumption of processed and energy-dense foods, declining physical activity levels, and rising sedentary behaviors, has contributed to the increasing prevalence of obesity and related metabolic disorders [42]. Additionally, the rapid urbanization seen in many regions during this period may have further elevated CVD mortality risk [43].

By 2032, total CVD deaths due to high BMI are projected to rise ∼33 %, though ASMR may slightly decline if healthcare improves. Regions like North Africa and the Middle East, South Asia, Southeast Asia, East Asia, Oceania, and Sub-Saharan Africa face increasing ASMRs, underscoring urgent intervention needs. Urbanization, dietary changes, physical inactivity, and weak health infrastructure drive these trends [44,45]. If current trends persist, the rising burden of high BMI-related CVD mortality could overwhelm already fragile healthcare systems in low- and middle-income countries. Preventive strategies, such as restricting unhealthy food marketing to children, expanding digital health tools, and implementing large-scale obesity prevention programs, could help mitigate future mortality trends. The projections in this study do not account for the potential impact of novel weight loss therapies, lipid-lowering treatments, or antihypertensive medications and procedures on obesity-related cardiovascular diseases. As these interventions become more widely used, they may reduce the burden of CVD related to high BMI, and future studies should explore the effects of these treatments on long-term mortality trends.

The clinical implications of our findings include the need to address age, gender, and regional disparities in high BMI-related cardiovascular mortality. Older adults, particularly those over 70, continue to experience the highest mortality, but the rising burden among younger individuals aged 15 to 49 highlights the importance of early prevention. Gender differences were also evident, with females experiencing more absolute deaths but showing greater improvements in mortality rates over time compared to males. Additionally, regional disparities tied to socio-demographic development were observed, with high-middle SDI regions carrying the highest mortality burden and low-middle SDI regions showing the fastest increase. These findings underscore the importance of implementing tailored interventions that consider age, gender, and regional context in order to reduce obesity-related cardiovascular deaths.

This study has several limitations. Data quality varies across regions, particularly in low-income and conflict-affected areas, which may lead to underreporting or misclassification of cardiovascular deaths. The GBD methodology is based on modeling assumptions that may not fully capture regional variations. Additionally, we were unable to adjust for important confounding factors such as smoking, hypertension, and type 2 diabetes, which are closely linked to both high BMI and cardiovascular outcomes. A further limitation is the inability to account for the effects of the COVID-19 pandemic. Individuals with obesity were more vulnerable to severe COVID-19 illness and death, and pandemic-related disruptions to healthcare access, preventive services, and lifestyle behaviors may have affected both BMI and cardiovascular mortality during 2019–2021.

## 5 Conclusion

Although high-BMI attributable CVD ASMR has slightly declined overall, the absolute number of deaths continues to rise, particularly in low to middle SDI regions. While mortality from traditional atherosclerotic diseases has decreased, conditions like HHD and AF/AFL are on the rise. Projections for 2032 indicate a continued increase in the absolute number of deaths, while global ASMR is expected to decrease slightly. These trends highlight the urgent need for global obesity prevention and targeted public health interventions to address the rising CVD burden attributed to high BMI.

## Funding

None

## CRediT authorship contribution statement

**Parisa Fallahtafti:** Writing – original draft, Visualization, Data curation, Conceptualization. **Hamidreza Soleimani:** Writing – review & editing, Writing – original draft, Supervision, Methodology, Formal analysis, Conceptualization. **Shaghayegh Khanmohammadi:** Writing – review & editing. **Amirhossein Habibzadeh:** Writing – original draft, Data curation. **Morvarid Taebi:** Visualization, Data curation. **Alireza Azarboo:** Writing – original draft. **Amirhossein Shirinezhad:** Writing – original draft. **Aysan Valinejad:** Writing – review & editing. **Michael J. Blaha:** Writing – review & editing, Supervision. **Sadeer Al-Kindi:** Writing – review & editing, Supervision. **Khurram Nasir:** Writing – review & editing, Supervision.

## Declaration of competing interest

The authors declare that they have no known competing financial interests or personal relationships that could have appeared to influence the work reported in this paper.

## Appendix Supplementary materials

Image, application 1
