# Supplementary material for: Global trends in cardiovascular mortality attributable to high body mass index: 1990–2021 analysis with future projections
Source: Am J Prev Cardiol. 2025 Oct 7;24:101326. doi: 10.1016/j.ajpc.2025.101326 (PMC12547811; doi:10.1016/j.ajpc.2025.101326)

Supplementary Materials

Global Trends in Cardiovascular Mortality Attributable to High Body Mass Index: 1990–2021 Analysis with Future Projections

Tables *2*

Supplementary Table 1. Mortality Burden of Cardiovascular Disease Attributable to High Body Mass Index in 204 Countries and Territories in 1990 and 2021, and its Temporal Trends 2

Supplementary Table 2. Wald Test Results for Net Drift, Deviations, and Relative Risk in Mortality Trends: Sex-Specific and Combined Analysis 10

Supplementary Table 3. Projected Death Counts and Mortality Rates for Cardiovascular Disease Attributable to High Body Mass Index (2022-2032) by Sex 11

Supplementary Table 4. Projected Death Counts and Mortality Rates for Cardiovascular Disease Attributable to High Body Mass Index (2022-2032) by Super region 12

Supplementary Table 5. Sensitivity Analysis of Projected Cardiovascular Disease Mortality Attributable to High Body Mass Index (2022–2032) under Different Overdispersion and Parameter Assumptions 13

Figures *14*

Supplementary Figure 1. Proportion of Cardiovascular Deaths attributable to High Body Mass Index by Age Group (1990–2021) 14

Supplementary Figure 2. Number of Cardiovascular Disease Deaths Attributable to High Body Mass Index in Super-regions (1990–2021) 15

Supplementary Figure 3. Age-standardized Mortality Rate (ASMR) of Cardiovascular Disease Attributable to high Body Mass Index in Super-regions (1990–2021) 16

Supplementary Figure 4. Sensitivity Analysis of projected Cardiovascular Disease Mortality Attributable to High Body Mass Index (2022–2032) under varying Overdispersion Assumptions 17

Supplementary Figure 5. Sensitivity Analysis of projected Cardiovascular Disease Mortality Attributable to High Body Mass Index (2022–2032) under varying Rate Assumptions 18

Supplementary Figure 6. Sensitivity Analysis of projected Cardiovascular Disease Mortality attributable to High Body Mass Index (2022–2032) under varying Shape Assumptions 19

Tables

Supplementary Table 1. Mortality Burden of Cardiovascular Disease Attributable to High Body Mass Index in 204 Countries and Territories in 1990 and 2021, and its Temporal Trends

Country	Total death counts			Age-standardized mortality rates		
	1990, N (95% UI)	2021, N (95% UI)	Percentage change, %, (95% CI)	1990, Rate per 100,000 (95% UI)	2021, Rate per 100,000 (95% UI)	Percentage change, %, (95% CI)
**Afghanistan**	4921.83 (2610.63–7585.75)	7287.01 (4030.39–11249.33)	48.05 (10–103.11)	73.16 (40.85–110.4)	77.4 (43.01–117.53)	5.79 (-21–39.54)
**Albania**	626.19 (285.33–1051.7)	1433.36 (613.82–2514.3)	128.9 (81–179.27)	35.28 (16.8–60.13)	34.67 (14.73–60.86)	-1.72 (-23–20.38)
**Algeria**	5034.71 (3193.81–7591.91)	17417.1 (9851.02–25853.33)	245.94 (171–341.86)	54.06 (32.76–81.27)	63.56 (35.44–96.75)	17.58 (-8–47.04)
**American Samoa**	12.79 (5.55–19.71)	29.54 (13.63–46.59)	131.01 (90–179.61)	53.91 (24.88–83.73)	62.14 (28.74–98.01)	15.27 (-5–38.15)
**Andorra**	7.72 (3.78–13.04)	17.77 (9.18–30.74)	130.2 (49–256.6)	15.85 (8.04–26.46)	10.06 (5.32–17.46)	-36.5 (-57–-4.98)
**Angola**	843.49 (529.78–1224.66)	3370.41 (2089.52–4921.13)	299.58 (166–553.82)	24.48 (15.52–34.89)	33.68 (21.7–49.6)	37.58 (-8–113.07)
**Antigua and Barbuda**	17.36 (11.26–25.29)	37.41 (26.17–50.65)	115.47 (85–150.29)	31.89 (20.82–46.54)	38.71 (25.36–53.21)	21.38 (3–43.76)
**Argentina**	10299.72 (5358.07–16395.45)	12567.04 (7183.34–18785.3)	22.01 (2–53.05)	33.64 (17.66–53.41)	21.91 (12.52–32.76)	-34.87 (-44–-20.79)
**Armenia**	1285.04 (606.57–2051.86)	1840.39 (915.62–2917.76)	43.22 (26–65.87)	53.16 (25.61–85.54)	42.83 (21.23–67.74)	-19.44 (-29–-7.98)
**Australia**	4595.23 (1878.73–7853.4)	5582.05 (2643.03–9273.9)	21.47 (4–47.21)	24.2 (9.97–41.62)	11.05 (5.14–18.26)	-54.36 (-61–-45.78)
**Austria**	3133.02 (1491.6–5201.15)	3506.03 (1952.68–5551.36)	11.91 (-18–66.25)	26.05 (12.29–42.94)	15.66 (8.84–24.96)	-39.9 (-53–-14.23)
**Azerbaijan**	2804.18 (1355.76–4489.4)	5267.96 (2453.61–8486.67)	87.86 (58–117.47)	61.52 (30.7–98.77)	60.05 (28.53–95.94)	-2.39 (-19–13.13)
**Bahrain**	100.6 (49.42–159.41)	312.83 (160.33–476.99)	210.96 (161–272.39)	70.84 (38.06–113.08)	51.28 (28–78.1)	-27.61 (-39–-11.61)
**Bangladesh**	2481.06 (1633.34–3544.05)	13717.95 (7248.92–21908.65)	452.91 (240–714.7)	5.57 (3.55–8.16)	10.49 (5.69–17.11)	88.32 (13–184.66)
**Barbados**	77.2 (42.19–123.16)	134.26 (73.72–208.99)	73.92 (37–114.91)	26.76 (14.72–42.45)	26.05 (14.34–40.57)	-2.66 (-23–21.47)
**Belarus**	5935.49 (2189.06–10245.38)	10283.4 (3685.32–17907.74)	73.25 (42–110.23)	47.6 (17.47–82.12)	63.39 (22.72–110.94)	33.19 (9–63)
**Belgium**	2463.61 (980.63–4136.65)	2080.1 (986.72–3486.12)	-15.57 (-30–8.16)	16.06 (6.36–26.89)	7.54 (3.41–12.72)	-53.06 (-59–-42.75)
**Belize**	27.03 (15.41–40.97)	92.08 (59.35–129.18)	240.63 (184–322.13)	29.02 (16.6–43.9)	32.49 (20.71–45.47)	11.95 (-7–41.56)
**Benin**	330.82 (194.19–490.98)	1104.51 (631.93–1685)	233.87 (132–352.56)	17.11 (9.86–25.92)	22.55 (12.86–34.76)	31.83 (-8–78.16)
**Bermuda**	23.6 (10.66–39.19)	31.02 (16.03–50)	31.41 (9–73.8)	39.84 (18.21–66.52)	21.19 (11.09–34.08)	-46.81 (-56–-31.05)
**Bhutan**	38.74 (19.99–64.45)	111.92 (60.41–183.85)	188.92 (109–318.47)	16.17 (8.47–25.95)	18.85 (10.45–30.81)	16.57 (-16–70.69)
**Bolivarian Republic of Venezuela**	3755.49 (2289.99–5642.34)	12010.17 (6351.14–18993.37)	219.8 (141–306.95)	41.19 (25.83–61.47)	41.27 (21.74–65)	0.2 (-25–26.53)

**Bosnia and Herzegovina**	1348.1 (600.24–2195.76)	2146.68 (1025.39–3528.71)	59.24 (26–102.28)	36.17 (16.61–58.57)	33.94 (16.19–55.69)	-6.18 (-26–18.39)
**Botswana**	157.42 (99.58–239.67)	495.19 (314.42–704.52)	214.57 (115–404.81)	33.95 (21.03–52.5)	41.74 (25.88–60.31)	22.94 (-17–94.27)
**Brazil**	25484.56 (14003.69–39112.19)	53384.28 (30139.73–82272.21)	109.48 (87–134.01)	30.17 (17.33–46.18)	21.54 (12.2–33.06)	-28.61 (-35–-21.42)
**Brunei Darussalam**	15.35 (8.53–23.01)	51.71 (24.74–77.88)	236.82 (156–319.83)	14.21 (8.22–21.06)	14.49 (7.73–21.72)	2 (-21–28.16)
**Bulgaria**	8551.11 (4337.09–13568.62)	14847.47 (9561.06–21083.6)	73.63 (31–175.4)	86.8 (43.51–137.45)	105.44 (67.21–150.34)	21.48 (-8–86.34)
**Burkina Faso**	392.29 (268.22–555.1)	1268.81 (815.65–1871.83)	223.43 (124–354.51)	9.54 (6.43–13.74)	14.44 (9.29–21.24)	51.34 (6–110.73)
**Burundi**	283.65 (100.13–452.12)	566.53 (370.38–817.12)	99.73 (30–345.89)	13.69 (4.61–22)	13.54 (8.31–20.32)	-1.07 (-35–98.79)
**Cambodia**	460.24 (270.26–669.83)	1491.31 (941.96–2163.09)	224.03 (118–391.32)	10.5 (6.18–15.25)	12.66 (8.01–18.76)	20.59 (-18–83.4)
**Cameroon**	1197.24 (712.81–1770.03)	4906.53 (2558.26–8105.31)	309.82 (184–480.89)	29.33 (17.82–42.71)	43.44 (22.88–69.99)	48.14 (3–112.86)
**Canada**	7354.43 (3009.15–12550.16)	8709.01 (4047.81–14159.08)	18.42 (4–43.53)	22.94 (9.38–39.2)	11.26 (5.28–18.11)	-50.91 (-57–-41.26)
**Central African Republic**	243.23 (122.18–365.49)	730.29 (408.09–1136.97)	200.24 (107–329.05)	24.16 (12.24–36.72)	37.4 (21.63–57.56)	54.82 (9–122.15)
**Chad**	424.68 (253.98–624.36)	1206.35 (690.95–1811.4)	184.06 (94–296.69)	15.97 (9.38–23.54)	22.43 (12.96–34.38)	40.43 (-1–92.23)
**Chile**	2476.01 (1274.74–4024.43)	4554.06 (2567.59–6903.52)	83.93 (53–142.75)	26.97 (13.85–43.64)	17.47 (9.88–26.51)	-35.24 (-45–-17.13)
**China**	88033.66 (61877.55–122803.5)	354345.48 (188060.3–564964.38)	302.51 (134–470.61)	13.73 (8.44–19.94)	18.8 (10.07–29.43)	36.95 (-20–102.23)
**Colombia**	4548.29 (2852.59–6524.31)	9800.94 (5126.68–15981.11)	115.49 (66–166.96)	28.32 (17.77–40.26)	17.33 (9.09–28.24)	-38.81 (-54–-22.9)
**Commonwealth of the Bahamas**	73.39 (52.62–100.93)	212.87 (148.57–290.19)	190.07 (127–257.27)	48.45 (34.21–66.82)	55.22 (37.31–76.19)	13.97 (-10–40.75)
**Comoros**	36.8 (21.16–54.34)	119.93 (73.34–178.82)	225.88 (119–398.29)	21.93 (12.77–32.41)	27.56 (16.95–41.97)	25.68 (-14–85.03)
**Congo**	377.33 (233.53–554.34)	1229.64 (773.67–1844.91)	225.88 (126–375.04)	38.25 (24.17–55.67)	51.93 (33.14–76.2)	35.76 (-4–98.79)
**Cook Islands**	12.12 (8.62–16.18)	16.57 (11.23–22.8)	36.73 (6–73.58)	100.7 (69.39–137.32)	66.23 (44.67–91.69)	-34.23 (-49–-17.74)
**Costa Rica**	341.57 (181.87–535.69)	894.2 (508.68–1372.51)	161.79 (122–208.85)	20.33 (10.88–31.84)	15.83 (9.02–24.28)	-22.14 (-33–-8.82)
**Croatia**	3103.29 (1579.15–5024.85)	3174.74 (1616.14–5061.05)	2.3 (-12–17.82)	57.92 (29.84–93.58)	33.21 (16.9–52.41)	-42.68 (-50–-34.34)
**Cuba**	2023.18 (879.67–3166.83)	5037.56 (2843.54–7747.74)	148.99 (98–247.65)	20.44 (8.84–32.21)	24.85 (14.06–38.27)	21.55 (-2–67.88)
**Cyprus**	185.95 (97.92–281.44)	329.98 (182.08–530.35)	77.45 (39–132.39)	33.35 (16.91–52.26)	20.38 (11.48–32.26)	-38.91 (-54–-17)
**Czech Republic**	8246.06 (2939.1–14213.97)	6702.76 (3445.04–10763.09)	-18.72 (-31–18.33)	60.71 (21.7–105.36)	29.29 (15.01–46.93)	-51.76 (-59–-30.43)
**Democratic People's Republic of Korea**	1197.14 (717.52–1869.8)	6060.01 (3456.25–10300.55)	406.21 (227–644.02)	10.21 (5.71–15.89)	21.43 (11.58–36.27)	109.98 (34–212.05)
**Democratic Republic of the Congo**	2834.21 (1661.25–4205.91)	12124.87 (7051.2–18147.1)	327.8 (198–510.05)	21.25 (12.62–31.56)	40.86 (25.03–61.85)	92.23 (35–169.37)

**Denmark**	1951.78 (775.63–3214.03)	1011 (451.51–1731.13)	-48.2 (-56–-36.14)	23.48 (9.24–38.77)	7.78 (3.4–13.34)	-66.86 (-71–-59.97)
**Djibouti**	15.16 (10.24–22.73)	83.07 (52.51–123.63)	447.87 (246–760.74)	13.81 (8.72–20.7)	15.75 (9.44–23.82)	14.09 (-22–76.89)
**Dominica**	33.19 (21.51–47.56)	44.82 (28.25–63.42)	35.03 (11–63.89)	57.78 (37.69–82.62)	56.65 (35.46–80.16)	-1.95 (-19–18.81)
**Dominican Republic**	707.4 (396.95–1104.45)	3077.69 (1605.81–4951.74)	335.07 (227–460.99)	20.01 (11.18–31.31)	30.75 (16.22–49.39)	53.66 (14–100.99)
**Ecuador**	1101.27 (671.31–1647.46)	3387.54 (1723.44–5414.13)	207.6 (133–295.82)	22.13 (13.57–32.59)	22.33 (11.78–35.43)	0.91 (-24–30.01)
**Egypt**	25431.16 (15001.45–37174.95)	69444.37 (36811.32–104589.06)	173.07 (110–230.74)	119.17 (71.4–174.46)	136.89 (75.82–203.74)	14.87 (-10–39.28)
**El Salvador**	621.59 (280.43–1022.24)	1437.7 (664.12–2383.52)	131.29 (86–189.52)	20.97 (9.64–34.18)	21.99 (10.06–36.35)	4.86 (-16–30.49)
**Equatorial Guinea**	73.2 (45.42–107.84)	207.28 (113.73–336.49)	183.16 (71–375.81)	41.01 (25.42–60.37)	48.71 (27.83–77.51)	18.77 (-26–89.98)
**Eritrea**	149.74 (81.3–219.64)	436.65 (271.79–641.13)	191.6 (86–381.19)	15.85 (8.99–24.15)	19.01 (11.63–28.96)	19.93 (-22–90.07)
**Estonia**	1264.53 (519.98–2063.1)	1799.42 (974.92–2498.04)	42.3 (-18–218.21)	64.27 (26.49–104.83)	56.17 (33.3–76.69)	-12.6 (-45–89.55)
**Ethiopia**	2996.84 (1850.31–4060.1)	4260.71 (2980.25–5822.41)	42.17 (-3–131.13)	16.68 (10.49–23.38)	10.69 (7.3–14.88)	-35.95 (-55–-3.28)
**Federated States of Micronesia**	40.71 (20.15–67.3)	72.26 (31.2–116.83)	77.48 (29–139.93)	81.12 (40.86–132.88)	93.54 (42.95–148.95)	15.31 (-14–54.62)
**Fiji**	250.55 (120.57–401.3)	582.01 (278.38–934.92)	132.29 (77–214.31)	68.08 (33.97–109.58)	81.65 (40.55–131.64)	19.93 (-6–58.13)
**Finland**	2244.06 (979.83–3718.22)	2899.43 (1583.65–4496.7)	29.2 (-4–100.68)	31.51 (13.7–51.93)	18.97 (10.51–29.11)	-39.78 (-52–-9.79)
**France**	10174.33 (5097.37–15871.41)	13496.74 (6757.31–21290.52)	32.65 (7–63.25)	11.84 (6.01–18.4)	7.24 (3.79–11.64)	-38.85 (-48–-28.4)
**Gabon**	248.04 (162.63–350.37)	523.45 (320.84–815.33)	111.03 (41–193.21)	47.83 (31.05–70.18)	59.94 (36.34–91.96)	25.32 (-14–72.37)
**Georgia**	3385.22 (1411.57–5529.18)	3143.69 (1897.96–4603.21)	-7.13 (-29–60.62)	57.19 (23.92–94.22)	50.13 (30.52–73.63)	-12.35 (-31–44.56)
**Germany**	45727.45 (22690.27–73251.37)	40351.46 (21616.95–62483.61)	-11.76 (-33–21.73)	35.17 (17.38–56.62)	17.25 (9.39–27.04)	-50.94 (-60–-36.15)
**Ghana**	1314.39 (839.62–1928.19)	5042.42 (2823.04–7997.45)	283.63 (167–457.31)	21.5 (13.99–31.16)	33.09 (18.22–51.41)	53.92 (8–122.09)
**Greece**	3322.31 (1438.11–5533.72)	5483.58 (2863.36–8806.98)	65.05 (29–128.44)	22.93 (9.82–38.5)	19.25 (10.03–30.83)	-16.04 (-31–7.85)
**Greenland**	13.76 (5.72–22.86)	13.41 (5.75–22.07)	-2.53 (-19–16.3)	45.35 (19.24–76.23)	21.38 (9.27–35.94)	-52.86 (-60–-44.11)
**Grenada**	21.05 (12.01–31.18)	36.73 (23.86–52.39)	74.53 (44–110.36)	29.8 (16.83–44.39)	34.69 (22.2–49.81)	16.43 (-5–45.31)
**Guam**	37.47 (22.5–54.52)	74.57 (37.17–114.63)	99 (51–140.61)	54.87 (34.19–80.19)	35.82 (17.76–54.91)	-34.71 (-53–-18.57)
**Guatemala**	657.81 (306.88–1073.01)	1857.07 (835.55–3134.63)	182.31 (138–223.74)	21.96 (10.87–35.3)	18.87 (8.73–31.27)	-14.07 (-26–-1.67)
**Guinea**	569.75 (351.42–841.63)	1371.19 (836.31–2008.64)	140.67 (56–268.52)	17.78 (10.84–26.14)	25.48 (15.42–37.28)	43.33 (-5–115.8)
**Guinea-Bissau**	104.63 (57.02–155.26)	278 (159.47–416.34)	165.7 (93–290.24)	27.11 (15.88–40.92)	39.82 (23.71–59.02)	46.91 (7–109.67)
**Guyana**	240.53 (158.99–339.62)	367.21 (229.31–539.85)	52.67 (15–96.32)	65.87 (43.37–92.64)	60.35 (38.26–88.11)	-8.38 (-30–16.33)
**Haiti**	625.71 (341.54–967.02)	1968.13 (1057.96–3099.73)	214.54 (112–337.41)	20.12 (11.26–30.57)	27.78 (15.11–43.82)	38.06 (-7–91.63)

**Honduras**	471.83 (282.26–719.81)	2512.28 (1388.46–3788.16)	432.46 (326–570.78)	25.34 (15.46–38.55)	44.96 (25.8–68.27)	77.45 (43–120.33)
**Hungary**	9567.88 (4808.75–15031.18)	10073.44 (5953.51–15083.17)	5.28 (-12–35.97)	68.84 (35.43–108.14)	48.57 (28.75–72.82)	-29.44 (-40–-11.46)
**Iceland**	72.09 (29.5–123.42)	76.63 (35.09–131.05)	6.3 (-10–36.79)	24.41 (9.95–41.78)	11.53 (5.33–19.59)	-52.75 (-59–-40.5)
**India**	29063.04 (17212.21–42605.28)	161910.58 (87259.37–241010.54)	457.1 (316–618.65)	6.47 (3.82–9.38)	14.1 (7.78–21.1)	117.83 (64–183.79)
**Indonesia**	8774.44 (5723.35–12099.53)	45829.84 (27573.29–67711.64)	422.31 (264–642.14)	8.99 (5.56–12.56)	19.63 (12.14–28.61)	118.26 (53–208.8)
**Iraq**	5724.38 (2769.72–9423.18)	16063.67 (7377.29–26244.76)	180.62 (112–279.76)	74.18 (36.23–121.96)	79.95 (39.21–128.73)	7.77 (-16–41.53)
**Ireland**	1145.82 (463.72–1957.23)	854.05 (376.61–1413.48)	-25.46 (-37–-10.42)	28.94 (11.84–49.38)	10.29 (4.53–17.01)	-64.45 (-70–-57.6)
**Islamic Republic of Iran**	9100.45 (5517.89–13043.1)	30813.6 (18213.82–44975.67)	238.59 (174–303.76)	40.83 (24.73–59.3)	44.1 (26.17–64.64)	8.01 (-12–26.92)
**Israel**	1158.8 (509.53–1904.43)	998.86 (462.92–1683.22)	-13.8 (-28–4.38)	25.15 (11.06–41.33)	7.27 (3.36–12.23)	-71.09 (-76–-66.01)
**Italy**	16725.88 (8879.62–25684.55)	28884.02 (14703.39–44347.63)	72.69 (16–148.58)	19.16 (10.09–29.6)	14.63 (7.9–22.04)	-23.64 (-43–2.62)
**Jamaica**	519.2 (356.63–724.09)	1023.19 (620.25–1544.36)	97.07 (50–153.55)	28.63 (19.74–39.8)	31.22 (19.25–47.2)	9.02 (-17–42.24)
**Japan**	9721.01 (5582.01–14691.73)	14790.06 (7761.73–23482.27)	52.15 (24–81.51)	6.33 (3.62–9.66)	3.28 (1.7–5.11)	-48.24 (-58–-39.58)
**Jordan**	857.72 (502.67–1298.23)	3166.09 (1917.43–4753.31)	269.13 (196–385.83)	74.38 (43.55–110.78)	53.35 (32.6–80.19)	-28.26 (-43–-6.11)
**Kazakhstan**	5824.39 (2411.34–9719.07)	7300.74 (2990.49–12496.27)	25.35 (10–42.12)	50.43 (20.74–83.74)	49.66 (20.37–85.63)	-1.53 (-14–13)
**Kenya**	822.32 (574.56–1109.62)	4092.01 (2652.71–5867.75)	397.61 (269–556.07)	11.3 (7.42–16)	21.23 (13.56–31.18)	87.89 (41–144.66)
**Kingdom of Eswatini**	127.58 (77.91–184.38)	368.08 (210.63–572.98)	188.51 (98–309.7)	55.76 (33.09–80.92)	79.55 (46.79–121.87)	42.68 (0–101.1)
**Kiribati**	18.75 (7.35–31.3)	49.84 (19.02–83.65)	165.82 (99–261.11)	45.28 (18.72–76.27)	62.43 (24.3–103.85)	37.86 (6–83.18)
**Kuwait**	319.42 (195.02–458.91)	1155.48 (616.98–1778.58)	261.74 (184–336.27)	55.91 (34.61–80.98)	41.3 (22.79–63.61)	-26.13 (-42–-10.49)
**Kyrgyzstan**	1300.08 (505.23–2171.86)	2341.1 (1064.33–3730.39)	80.07 (51–126.48)	46.55 (18.11–77.95)	55.9 (26.3–89.69)	20.09 (0–51.54)
**Lao People's Democratic Republic**	366.01 (187.95–560.76)	974.3 (551.32–1541.5)	166.2 (70–338.93)	18.51 (9.56–28.5)	21.69 (12.73–34.04)	17.21 (-23–90.03)
**Latvia**	2111.86 (726.47–3690.71)	2053.18 (1073.69–3370.05)	-2.78 (-19–50.66)	60.64 (20.96–106.27)	46.62 (23.99–76.77)	-23.12 (-36–16.88)
**Lebanon**	1056.46 (517.65–1689.01)	1814.39 (937.88–2854.9)	71.74 (32–127.63)	53.4 (26.34–85.52)	28.16 (14.57–44.14)	-47.27 (-59–-31.67)
**Lesotho**	260.35 (166.16–377.99)	644.06 (387.16–947.04)	147.39 (62–262.88)	34.87 (22.15–51.48)	68.95 (42.23–101.9)	97.76 (35–187.23)
**Liberia**	317.71 (204.06–476.77)	883.56 (497.68–1373.5)	178.1 (90–299.08)	28.46 (18.22–42.17)	43.08 (24.83–67.84)	51.39 (6–117.17)
**Libya**	679.24 (355.55–1041.54)	3377.86 (1845.88–5279.45)	397.3 (261–584.82)	37.7 (19.7–57.59)	69.82 (37.75–108.86)	85.22 (35–154.1)

**Lithuania**	2264.17 (863.03–3891.51)	2785.18 (1325.08–4598.95)	23.01 (5–56.79)	51.49 (19.64–88.24)	43.41 (20.77–71.64)	-15.69 (-27–5.96)
**Luxembourg**	129.11 (52.95–214.43)	132.06 (67.7–212.54)	2.28 (-18–49.41)	24.5 (10.27–40.54)	10.95 (5.59–17.82)	-55.32 (-63–-38.1)
**Madagascar**	1019.47 (718.84–1457.09)	3418.69 (2114.79–4905.8)	235.34 (127–382.61)	22.53 (15.15–32.96)	36.15 (22.41–52.71)	60.42 (11–134.28)
**Malawi**	402.27 (241.99–591.65)	1419.04 (890.96–2142.71)	252.75 (145–411.54)	12.35 (7.15–18.29)	21.4 (13.45–31.33)	73.27 (23–146.96)
**Malaysia**	1535.99 (692.69–2426.96)	5714.47 (2359.09–9116.52)	272.04 (212–327.19)	16.41 (7.6–25.66)	20.79 (8.64–33.46)	26.65 (4–48.62)
**Maldives**	14.24 (7.09–22.23)	34.78 (17.69–56.22)	144.3 (79–241.75)	14.07 (7.14–21.59)	9.58 (5.22–15.12)	-31.92 (-51–-3.23)
**Mali**	598.09 (339.32–877.14)	1411.78 (831.55–2111.32)	136.05 (70–221.15)	15.97 (9.08–23.54)	16.68 (9.9–24.95)	4.5 (-25–44.14)
**Malta**	92.95 (40.38–148.42)	141.41 (72.08–230.83)	52.13 (24–100.15)	22.81 (9.89–36.21)	13.25 (6.78–21.56)	-41.93 (-52–-26.49)
**Marshall Islands**	12.78 (6.28–20.28)	34.32 (14.98–56.44)	168.6 (102–242.09)	76.78 (39.32–121.63)	93.44 (43.39–152.66)	21.69 (-7–51.34)
**Mauritania**	403.33 (231.73–616.58)	893.79 (495.87–1409.02)	121.6 (59–220.22)	43.34 (25.44–65.39)	46.37 (25.48–72.47)	6.98 (-23–50.36)
**Mauritius**	253.4 (164.99–359.41)	466.71 (293.48–661)	84.18 (54–119.86)	35.85 (22.99–50.31)	26.81 (16.78–37.63)	-25.24 (-37–-11.1)
**Mexico**	7956.88 (4263.39–12486.25)	30430.91 (15246.28–48705.01)	282.45 (224–338.87)	21.28 (11.94–33.46)	25.52 (12.93–40.91)	19.89 (0–38.67)
**Mongolia**	416.96 (167.3–708.65)	665.12 (244.16–1140.91)	59.52 (34–90.21)	42.98 (17.86–72.83)	33.44 (12.85–56.55)	-22.18 (-34–-6.86)
**Montenegro**	239.39 (97.44–405.33)	527.48 (215.26–894.66)	120.35 (89–155.36)	39.92 (16.33–67.31)	59.9 (25.05–101.05)	50.04 (29–73.74)
**Morocco**	7085.56 (4032.35–10838.97)	21503.97 (12262.55–32579)	203.49 (132–270.74)	52.93 (30.49–80.07)	68.7 (39.06–103.35)	29.8 (0–56.25)
**Mozambique**	853.18 (550.16–1204.97)	2923.45 (1786.88–4335.76)	242.65 (128–404.09)	16.8 (10.59–23.88)	29.8 (18.16–45.4)	77.36 (16–156)
**Myanmar**	3914.93 (2222.04–6079.09)	7592.74 (4492.76–11832.84)	93.94 (31–197.79)	16.8 (9.72–25.42)	15.81 (9.53–24.52)	-5.89 (-36–40.81)
**Namibia**	189.33 (127.74–273.17)	623.02 (377.19–933.34)	229.07 (121–369.06)	35.48 (23.5–54.21)	55.39 (32.51–83.63)	56.11 (6–121.08)
**Nepal**	614.09 (351.84–966.04)	2682.76 (1510.33–4182.91)	336.87 (189–575.3)	6.34 (3.82–9.74)	11.67 (6.7–18.21)	84.14 (23–173.16)
**Netherlands**	3589.03 (1430.69–6133.65)	3109.98 (1479.72–5244.12)	-13.35 (-28–13.22)	17.89 (7.12–30.7)	7.99 (3.75–13.45)	-55.32 (-62–-42.8)
**New Zealand**	1138.77 (479.39–1890.23)	1197.93 (519.91–2006.94)	5.19 (-9–16.76)	29.52 (12.44–48.95)	13.43 (5.84–22.27)	-54.51 (-61–-49.94)
**Nicaragua**	282.54 (152.66–435.63)	950.54 (479.18–1535.33)	236.42 (180–305.82)	19.84 (11.1–30.62)	21.46 (11.31–34.85)	8.17 (-11–30.84)
**Niger**	347.69 (195.97–513.58)	1201.22 (653.63–1823.14)	245.49 (135–436.62)	13.82 (7.58–21.56)	15.7 (8.75–24)	13.58 (-23–77.16)
**Nigeria**	8007.48 (5305.19–11469.35)	21323.94 (11781.89–30951.38)	166.3 (74–280.33)	20.64 (13.17–29.8)	27.66 (15.36–40.37)	34.02 (-9–87.25)
**Northern Mariana Islands**	8.06 (2.82–14.03)	23.76 (9.85–37.97)	194.66 (128–296.76)	37.23 (13.81–63.83)	46.55 (19.36–75.04)	25.02 (0–55.97)
**Norway**	1546.78 (619.58–2590.96)	797.58 (378.32–1330.6)	-48.44 (-56–-35.43)	21.81 (8.64–36.35)	6.88 (3.16–11.44)	-68.48 (-72–-61.56)
**Oman**	345.75 (191.07–549.47)	1080.41 (609.83–1578.02)	212.49 (115–338.48)	54.25 (30.08–83.08)	66.23 (39.65–96.63)	22.07 (-15–72.55)

**Pakistan**	6507.23 (3643.21–9958.61)	32442.25 (17622.02–50731.25)	398.56 (258–590.77)	12.16 (6.79–18.49)	27.56 (15.71–42.27)	126.52 (65–210.26)
**Palestine**	609.87 (350.31–987.32)	1394.86 (777.19–2062.61)	128.71 (78–199.14)	80.62 (45.54–128.29)	70.67 (38.68–104.4)	-12.35 (-31–14.3)
**Panama**	286.81 (130.02–489.54)	878.01 (439.27–1398.74)	206.13 (141–291.93)	20.71 (9.36–35.31)	19.26 (9.65–30.67)	-6.99 (-27–18.76)
**Papua New Guinea**	385.45 (194.13–677.19)	1396.83 (664.1–2526.38)	262.39 (140–430.67)	19.34 (10.24–33.1)	23.67 (11.51–41.5)	22.39 (-16–74.38)
**Paraguay**	509.48 (271.43–820.35)	1579.53 (885.45–2578.22)	210.03 (132–297.81)	23.86 (12.69–38.26)	28.18 (16.08–45.65)	18.09 (-12–51.15)
**Peru**	1496.09 (749.33–2473.09)	3506.94 (1688.07–5759.76)	134.41 (71–202.63)	12.7 (6.64–20.8)	10.3 (5.01–16.88)	-18.93 (-41–4.9)
**Philippines**	4845.96 (3437.12–6607.48)	22022.42 (13830.92–32092)	354.45 (268–457.74)	17.08 (11.89–23.3)	27.32 (17.66–39.36)	59.95 (31–96.19)
**Plurinational State of Bolivia**	659.49 (345.09–1035.4)	1756.14 (896.99–2878.75)	166.29 (106–259.92)	21.85 (11.5–33.89)	21.11 (11.02–34.13)	-3.39 (-24–30.68)
**Poland**	21389.35 (9665.43–35283.53)	22867.35 (12359.79–35756.09)	6.91 (-10–37.59)	51.45 (23.4–84.81)	29.87 (16.33–46.6)	-41.94 (-50–-26.15)
**Portugal**	2745.78 (1120.43–4848.08)	3269.24 (1628.9–5321.51)	19.06 (-9–81.06)	21.56 (8.8–38.2)	11.12 (5.54–18.37)	-48.43 (-58–-28.51)
**Principality of Monaco**	16.35 (6.56–28.63)	16.8 (8.17–28.52)	2.8 (-22–49.59)	21.7 (8.47–37.35)	14.5 (7.23–24.55)	-33.19 (-49–-3.3)
**Puerto Rico**	1079.57 (579.28–1697.43)	1474.84 (862.88–2188.39)	36.61 (11–79.18)	31.31 (16.67–49.42)	18.86 (11.06–28.03)	-39.78 (-51–-23.34)
**Qatar**	65.78 (30.86–105.63)	266.35 (124.76–422.94)	304.88 (204–453.93)	77.61 (39.38–125.78)	40.28 (20.11–63.35)	-48.11 (-60–-32.75)
**Republic of CÃ´te d'Ivoire**	767.32 (471.21–1143.17)	3352.71 (1896.21–5183.47)	336.94 (213–523.99)	21.19 (13.29–31.2)	32.43 (19.26–48.88)	53.01 (8–116.38)
**Republic of Cabo Verde**	34.38 (21.73–49.41)	115.36 (67.45–181.35)	235.55 (130–377.36)	15.17 (9.6–21.47)	26.7 (15.64–41.79)	75.97 (20–148.19)
**Republic of Korea**	1737.09 (1067.88–2799.57)	4069.24 (2256.74–6551.62)	134.26 (52–264.18)	8.01 (4.4–12.57)	4.58 (2.51–7.38)	-42.82 (-60–-13.55)
**Republic of Moldova**	2293.64 (839.14–3835.69)	4094.39 (2248.64–6181.97)	78.51 (48–179.22)	61.33 (22.73–102.94)	67.87 (37.34–102.34)	10.65 (-9–73.06)
**Republic of Nauru**	6.55 (2.87–10.08)	9.8 (4.2–15.52)	49.54 (17–95.36)	121.29 (55.56–189.63)	151.95 (67.12–239.36)	25.27 (-1–60.42)
**Republic of Niue**	1.31 (0.66–2.06)	1.54 (0.74–2.46)	17.13 (-9–48.36)	60.23 (30–94.91)	73.04 (35.42–117.48)	21.27 (-6–53.35)
**Republic of Palau**	5.97 (2.17–10.29)	14.39 (5.77–24.36)	140.97 (85–214.46)	60.09 (22.42–101.93)	66.56 (26.83–112.21)	10.77 (-13–41.63)
**Republic of San Marino**	5.8 (2.94–9.33)	7.61 (3.65–13.19)	31.14 (-10–92.18)	15.73 (7.97–25.4)	7.47 (3.63–12.99)	-52.53 (-68–-30.61)
**Republic of the Gambia**	73.51 (45.13–107.81)	346.19 (197.93–541.38)	370.91 (231–559.77)	22.18 (13.78–32.9)	37.32 (21.48–58.15)	68.24 (19–135.48)
**Romania**	15985.74 (8967.27–24435.21)	21225.41 (12098.86–32265.96)	32.78 (10–58.88)	65.04 (36.11–101.1)	53.41 (30.45–81.59)	-17.88 (-29–-4.44)
**Russian Federation**	84806.4 (28547.62–145459.7)	119568.61 (47148.43–200548.71)	40.99 (19–66.44)	51.62 (17.2–88.71)	50.03 (19.69–84.12)	-3.07 (-19–14.42)
**Rwanda**	551.41 (211.95–801.13)	881.08 (498.85–1328.53)	59.79 (-1–175.5)	22.25 (8.9–32.96)	17.04 (8.72–26.23)	-23.38 (-51–24.3)
**Saint Kitts and Nevis**	15.65 (8.17–24.65)	23.38 (13.56–34.8)	49.37 (22–84.46)	43.5 (22.72–69.04)	38.85 (22.85–58.18)	-10.69 (-27–13.39)
**Saint Lucia**	26.56 (17.25–38.73)	62 (37.96–91.77)	133.43 (88–188.09)	34.27 (21.6–50.23)	26.65 (16.22–39.59)	-22.24 (-36–-5.51)
**Saint Vincent and the Grenadines**	20.42 (14.08–29.03)	52.85 (36.33–72.92)	158.76 (115–214.59)	30.43 (20.36–43.88)	40.31 (26.69–56.52)	32.43 (10–61.06)

**Samoa**	55.87 (27.6–86.15)	113.2 (52.4–177.67)	102.61 (60–163.51)	65.35 (33.72–101.09)	78.62 (36.96–123.33)	20.31 (-4–53.16)
**Sao Tome and Principe**	10.61 (6.33–16.17)	28.57 (14.14–44.86)	169.39 (100–249.09)	17.25 (10.41–26.18)	27.97 (14.41–44.55)	62.16 (24–108.22)
**Saudi Arabia**	3808.7 (2061.49–6036.7)	16354.68 (8852.23–24853.22)	329.4 (204–510.71)	71.22 (38.8–110.1)	88.75 (50.72–132.81)	24.62 (-6–70.86)
**Senegal**	667.04 (423.65–974.99)	2101.64 (1274.61–3170.7)	215.07 (133–318.9)	21.65 (13.69–31.12)	29.15 (17.76–42.98)	34.62 (0–80.72)
**Serbia**	5628.45 (2754.92–9061.18)	9172.7 (4762.14–14404.96)	62.97 (36–96.86)	63.38 (31.95–102.93)	53.49 (27.65–83.77)	-15.61 (-30–2.24)
**Seychelles**	26.55 (19.67–34.48)	48.9 (33.75–65.89)	84.22 (51–119.95)	47.16 (34.91–61.41)	45.22 (30.23–61.61)	-4.1 (-22–13.93)
**Sierra Leone**	325.26 (199.82–479.16)	931.01 (515.75–1438.94)	186.23 (109–294.71)	16.93 (10.13–24.99)	26.01 (15.02–40.43)	53.64 (14–113.26)
**Singapore**	253.96 (150.16–372.96)	649.32 (391.28–945.81)	155.68 (119–202.95)	11.82 (7.11–17.55)	7.61 (4.55–11.14)	-35.65 (-46–-23.89)
**Slovakia**	3825.34 (1618.9–6270.67)	4047.71 (1967.74–6618.12)	5.81 (-8–32.81)	65.84 (27.96–107.9)	42.45 (20.75–69.34)	-35.52 (-44–-19.64)
**Slovenia**	845.08 (444.73–1330.06)	1125.01 (574.51–1733.91)	33.13 (-10–102.22)	34.66 (18.13–54.68)	20.6 (10.74–31.47)	-40.56 (-57–-13.73)
**Socialist Republic of Viet Nam**	1704.93 (1071.4–2690.06)	7319.69 (4424.23–10881.77)	329.32 (147–583.32)	4.65 (2.86–7.52)	8.02 (4.71–11.99)	72.51 (3–168.61)
**Solomon Islands**	58.08 (25.96–102.66)	200.03 (82.25–346.95)	244.4 (139–423.75)	39.97 (19.03–70.29)	52.99 (23.12–91.39)	32.59 (-5–84.8)
**Somalia**	441.65 (240.73–664.08)	1181.98 (657.45–1786.11)	167.63 (80–304.42)	19.4 (11.25–28.08)	21.28 (11.74–33.02)	9.66 (-24–60.4)
**South Africa**	6452.43 (4348.9–8997.76)	20381.56 (13388.36–28358.41)	215.87 (179–249.29)	32.94 (21.49–46.09)	50.22 (32.69–71.03)	52.46 (34–70.9)
**South Sudan**	249.77 (135.1–392.16)	411.68 (242.93–621.82)	64.82 (11–152)	11.08 (6.07–17.65)	11.9 (6.82–17.72)	7.4 (-25–61.13)
**Spain**	9782.76 (4141.07–16853.16)	14098.47 (7283.47–22661.8)	44.12 (8–122.48)	18.52 (7.89–32.02)	11.33 (5.98–17.99)	-38.86 (-50–-13.4)
**Sri Lanka**	1476.4 (1030.39–2041.67)	3928.69 (1963.13–6627.32)	166.1 (63–288.46)	14.81 (9.94–20.58)	15.35 (7.59–26.19)	3.62 (-37–50.88)
**Sudan**	5955.9 (3495.12–9133.17)	14043.73 (7265.45–22196.52)	135.8 (72–216.33)	68.24 (39.9–102.05)	77.61 (41.81–123.47)	13.72 (-15–48.88)
**Suriname**	63.36 (37.91–94.83)	155.86 (84.36–243.73)	145.98 (84–214.56)	25.16 (15.24–37.65)	24.79 (13.39–38.41)	-1.46 (-26–25.81)
**Sweden**	3418.52 (1328.78–5815.73)	2892.94 (1488.14–4638.7)	-15.37 (-36–37.68)	21.39 (8.24–36.16)	10.8 (5.53–17.26)	-49.5 (-60–-20.8)
**Switzerland**	2143.35 (1091.73–3427.27)	2240.83 (1189.25–3452.31)	4.55 (-28–61.9)	19.57 (9.87–31.26)	9.27 (5.13–14.24)	-52.62 (-65–-29.05)
**Syrian Arab Republic**	3777.21 (1947.48–5774.53)	11961.56 (5701.28–19497.74)	216.68 (123–336.27)	77.69 (42.03–120.05)	108.59 (52.88–174.49)	39.76 (0–89.25)
**Taiwan (Province of China)**	1668.18 (1124.73–2363.74)	4253.73 (2587.11–6109.85)	154.99 (97–215.61)	12.67 (8.62–18.32)	9.76 (5.99–13.93)	-22.93 (-36–-7.72)
**Tajikistan**	1582.69 (848.75–2411.92)	2642.89 (1330.36–4234.18)	66.99 (32–110.61)	62.55 (33.82–94.78)	56.35 (28.76–89.25)	-9.91 (-29–14.77)
**Thailand**	1706.04 (800.1–2777.68)	7914.42 (3249.91–13818.03)	363.91 (226–559.1)	4.9 (2.37–7.98)	7.44 (3.04–12.91)	51.78 (8–113.14)
**The former Yugoslav Republic of Macedonia**	1059.97 (534.26–1659.47)	1792.3 (932.05–2893.81)	69.09 (41–106.02)	62.15 (33.08–96.71)	71.28 (36.34–114.36)	14.68 (-5–39.61)
**Timor-Leste**	15.16 (7.79–23.35)	81.38 (52.11–120.22)	436.63 (217–884.58)	6.11 (3.12–10)	9.97 (6.38–14.42)	63.24 (-2–194.31)

**Togo**	234.27 (149.02–345.54)	1128.7 (652.38–1698.36)	381.8 (235–562.24)	20.39 (13.16–29.85)	33.24 (19.05–50.18)	63.02 (15–122.39)
**Tokelau**	0.78 (0.41–1.31)	0.94 (0.44–1.53)	19.59 (-9–53.61)	60.11 (31.41–100.81)	64.08 (30.21–104.57)	6.6 (-18–36.66)
**Tonga**	19.76 (8.25–31.48)	35.78 (16.39–57.82)	81.07 (34–140.86)	34.98 (15.22–56.01)	44.68 (20.5–72.52)	27.74 (-4–69.71)
**Trinidad and Tobago**	381.39 (233.23–545.93)	619.3 (336.45–961.04)	62.38 (22–104.22)	49.88 (30.31–71.55)	32.69 (17.88–50.86)	-34.47 (-52–-17.19)
**Tunisia**	1653.13 (1032.78–2444.72)	6138 (3334.37–9622.44)	271.29 (165–442.51)	38.08 (23.22–57.2)	50.66 (28.02–79.79)	33.01 (-5–90.46)
**Turkey**	15845.77 (8984.29–24064.76)	38205.95 (21113.32–58421.62)	141.11 (88–202.96)	52.13 (29.51–78.69)	44.72 (24.91–68.28)	-14.21 (-33–6.17)
**Turkmenistan**	1075.35 (457.95–1753.37)	2428.87 (1027.38–4153.31)	125.87 (79–182.19)	61.09 (26.61–99.28)	65.82 (28.34–110.93)	7.73 (-14–33.22)
**Tuvalu**	5.17 (2.51–8.27)	8.81 (4.15–13.93)	70.23 (40–108.61)	74.44 (38.48–118.79)	85.21 (40.58–135.3)	14.47 (-6–39.58)
**Uganda**	630.3 (334.57–964.56)	1986.36 (1209.31–3051.63)	215.15 (107–385.3)	10.92 (5.48–16.6)	15.02 (8.77–23.72)	37.47 (-8–107.47)
**Ukraine**	36489.04 (13782.07–62000.12)	55754.73 (21966.21–94472.95)	52.8 (17–99.2)	54.53 (20.69–93.72)	71 (27.97–120.53)	30.21 (-2–69.84)
**United Arab Emirates**	228.5 (109.95–373.97)	1419.48 (731.83–2210.86)	521.22 (390–681.32)	55.78 (29.07–87.22)	63.75 (35.49–94.89)	14.28 (-9–41.96)
**United Kingdom of Great Britain and Northern Ireland**	28906.89 (11797.11–49193.26)	17668.13 (8378.58–29442.07)	-38.88 (-45–-25.49)	31.68 (12.93–53.61)	12.59 (6.03–20.81)	-60.27 (-64–-52.29)
**United Republic of Tanzania**	1894.27 (1136.7–2674.85)	6645.55 (4005.09–10075.73)	250.82 (143–401.68)	20.61 (11.64–31.19)	30.28 (18.43–46.13)	46.92 (1–111.26)
**United States of America**	99181.77 (47397–162300.23)	152511.93 (87452.64–228828.32)	53.77 (36–92.06)	31.01 (14.89–50.33)	25.66 (14.8–38.01)	-17.26 (-27–3.3)
**United States Virgin Islands**	38.57 (21.23–59.39)	51.47 (28.18–78.98)	33.42 (4–71.66)	53.11 (29.53–82.84)	30.75 (16.77–47.32)	-42.09 (-54–-25.03)
**Uruguay**	1083.95 (505.37–1779.01)	1219.26 (666.74–1861.79)	12.48 (-9–57.32)	28.24 (13.1–46.43)	19.9 (10.95–30.38)	-29.55 (-40–-6.68)
**Uzbekistan**	5478.09 (2345.24–8932.2)	14770.17 (6907.26–23809.64)	169.62 (128–220.57)	50.55 (21.69–82.53)	65.65 (31–107.66)	29.86 (11–54.62)
**Vanuatu**	28.27 (13.59–50.29)	108.48 (47.55–179.85)	283.79 (174–421.03)	42.98 (21.37–74.24)	58.45 (26.88–97.04)	35.99 (1–78.27)
**Yemen**	2085.78 (1248.43–3086.3)	8610.35 (4739.51–13218.37)	312.81 (187–505.85)	47.22 (29.16–69.36)	68.95 (39.36–107.54)	46.02 (1–113.35)
**Zambia**	549.55 (380.94–746.59)	2236.89 (1303.55–3367.06)	307.04 (161–498.35)	21.78 (14.58–30.75)	37.11 (21.87–55.1)	70.35 (12–141.26)
**Zimbabwe**	615.49 (417.64–834.11)	2728.71 (1614.07–4153.28)	343.34 (192–514.2)	17.7 (11.76–25.91)	44.4 (27.27–66.29)	150.8 (66–248.89)

UI: uncertainty intervals; CI: confidence interval; SDI: socio-demographic index

Supplementary Table 2. Wald Test Results for Net Drift, Deviations, and Relative Risk in Mortality Trends: Sex-Specific and Combined Analysis

Test conditions	df	Female		Male		Both	
		X^2	P-value	X^2	P-value	X^2	P-value
Net drift = 0	1	120.52	0	6.40	0.0114	12.61	0.0004
All age deviations = 0	14	4069.09	0	3037.73	0	4120.44	0
All period deviations = 0	4	28.18	0	4.83	0.3046	12.14	0.0163
All cohort deviations = 0	19	477.32	0	215.20	0	364.64	0
All period RR = 1	5	143.93	0	12.55	0.028	23.69	0.0002
All cohort RR = 1	20	1566.32	0	238.33	0	676.75	0
All local drifts = Net drift	16	466.02	0	205.31	0	356.01	0

df: degrees of freedom; X^2: Chi-square statistic; RR: relative risk.
P-value < 0.05 indicates significant deviation from the null hypothesis.

Supplementary Table 3. Projected Death Counts and Mortality Rates for Cardiovascular Disease Attributable to High Body Mass Index (2022-2032) by Sex

Year	Death count, N	Mortality rate (per 100,000)	Death count, N	Mortality rate (per 100,000)	Death count, N	Mortality rate (per 100,000)
	Global		Female		Male	
**2022**	1939329	22.47	1014154	21.19	936351	23.76
**2023**	1986942	22.39	1041171	21.15	959142	23.68
**2024**	2040339	22.32	1071656	21.13	984243	23.61
**2025**	2095311	22.25	1103169	21.10	1010128	23.55
**2026**	2150188	22.18	1134656	21.06	1036260	23.48
**2027**	2205154	22.13	1166106	21.04	1062610	23.44
**2028**	2264286	22.10	1200004	21.04	1090621	23.42
**2029**	2328613	22.08	1237127	21.05	1120789	23.41
**2030**	2395401	22.06	1275935	21.06	1152119	23.41
**2031**	2464183	22.05	1315942	21.07	1184514	23.42
**2032**	2535765	22.06	1357482	21.10	1218152	23.46

Supplementary Table 4. Projected Death Counts and Mortality Rates for Cardiovascular Disease Attributable to High Body Mass Index (2022-2032) by Super region

Year	Death count, N	Mortality rate (per 100,000)	Death count, N	Mortality rate (per 100,000)	Death count, N	Mortality rate (per 100,000)	Death count, N	Mortality rate (per 100,000)
	**Global**		**High income**		**Central Europe, Eastern Europe, and Central Asia**		**Latin America and Caribbean**	
**2022**	1939329	22.47	333525	13.88	333567	50.06	146687	22.92
**2023**	1986942	22.39	331891	13.59	335459	49.47	151218	22.85
**2024**	2040339	22.32	331236	13.33	338836	48.88	156124	22.79
**2025**	2095311	22.25	330837	13.07	343166	48.26	161216	22.72
**2026**	2150188	22.18	330350	12.84	348206	47.62	166411	22.67
**2027**	2205154	22.13	329535	12.62	353874	47.00	171651	22.62
**2028**	2264286	22.10	329093	12.41	361092	46.39	177224	22.58
**2029**	2328613	22.08	329358	12.21	370703	45.79	183231	22.56
**2030**	2395401	22.06	329993	12.02	382760	45.16	189539	22.55
**2031**	2464183	22.05	330846	11.85	397569	44.53	196095	22.55
**2032**	2535765	22.06	331728	11.70	415596	43.91	202856	22.57
	**North Africa and Middle East**		**South Asia**		**Southeast Asia, East Asia, and Oceania**		**Sub-Saharan Africa**	
**2022**	611506	67.44	434024	15.03	501198	18.40	134451	30.27
**2023**	639105	67.60	450526	15.16	523325	18.61	140908	30.56
**2024**	668970	67.77	468098	15.28	547464	18.83	147940	30.85
**2025**	700453	67.93	486462	15.41	572861	19.05	155428	31.14
**2026**	733146	68.09	505469	15.54	599047	19.28	163245	31.42
**2027**	766718	68.25	524990	15.66	625657	19.52	171322	31.70
**2028**	802168	68.42	545376	15.79	653565	19.77	179899	31.97
**2029**	840276	68.59	566932	15.92	683533	20.03	189182	32.25
**2030**	880513	68.77	589522	16.04	715181	20.29	199091	32.52
**2031**	922536	68.95	613176	16.17	748326	20.57	209526	32.80
**2032**	966004	69.15	637833	16.29	782790	20.86	220439	33.06

Supplementary Table 5. Sensitivity Analysis of Projected Cardiovascular Disease Mortality Attributable to High Body Mass Index (2022–2032) under Different Overdispersion and Parameter Assumptions

**Year**	**Overdispersion**		**Rate parameter**				**Shape parameter**			
	No Overdispersion	Overdispersion	0.000001	0.00001	0.01	0.1	0.25	0.5	2	5
**2022**	22.946	22.466	22.464	22.464	22.524	22.530	22.467	22.466	22.464	22.465
**2023**	23.021	22.388	22.384	22.384	22.514	22.540	22.391	22.390	22.385	22.381
**2024**	23.088	22.321	22.314	22.315	22.517	22.576	22.326	22.324	22.316	22.308
**2025**	23.070	22.251	22.241	22.242	22.489	22.535	22.257	22.255	22.244	22.233
**2026**	23.089	22.182	22.170	22.171	22.461	22.522	22.188	22.186	22.173	22.160
**2027**	23.151	22.128	22.114	22.115	22.450	22.508	22.136	22.133	22.119	22.103
**2028**	23.267	22.100	22.082	22.084	22.488	22.564	22.109	22.105	22.088	22.068
**2029**	23.361	22.082	22.062	22.063	22.533	22.638	22.092	22.088	22.068	22.044
**2030**	23.371	22.065	22.042	22.044	22.549	22.635	22.076	22.072	22.050	22.023
**2031**	23.441	22.054	22.030	22.032	22.580	22.678	22.067	22.062	22.038	22.010
**2032**	23.602	22.065	22.038	22.041	22.646	22.756	22.079	22.074	22.048	22.016

Figures

Supplementary Figure 1. Proportion of Cardiovascular Deaths attributable to High Body Mass Index by Age Group (1990–2021)

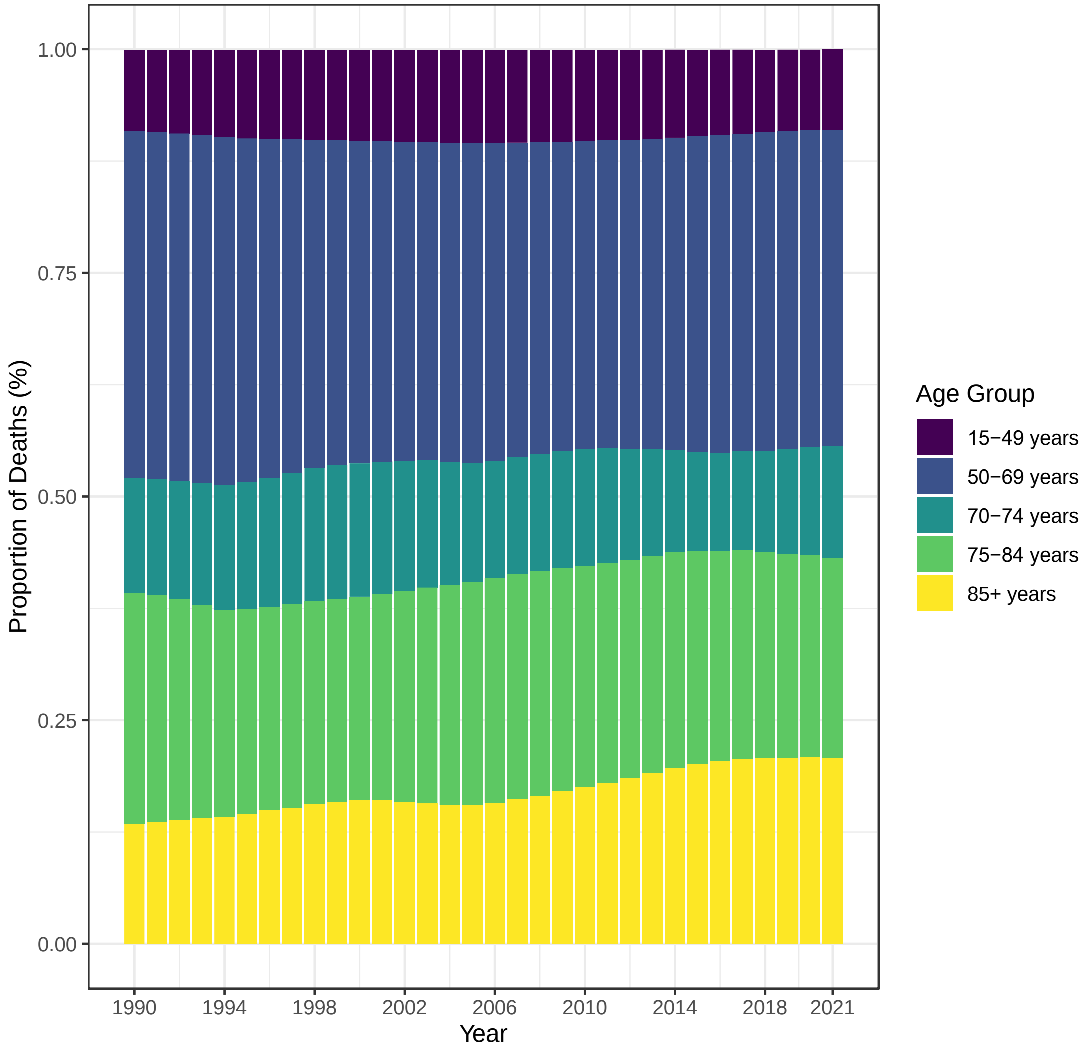

Supplementary Figure 2. Number of Cardiovascular Disease Deaths Attributable to High Body Mass Index in Super-regions (1990–2021)

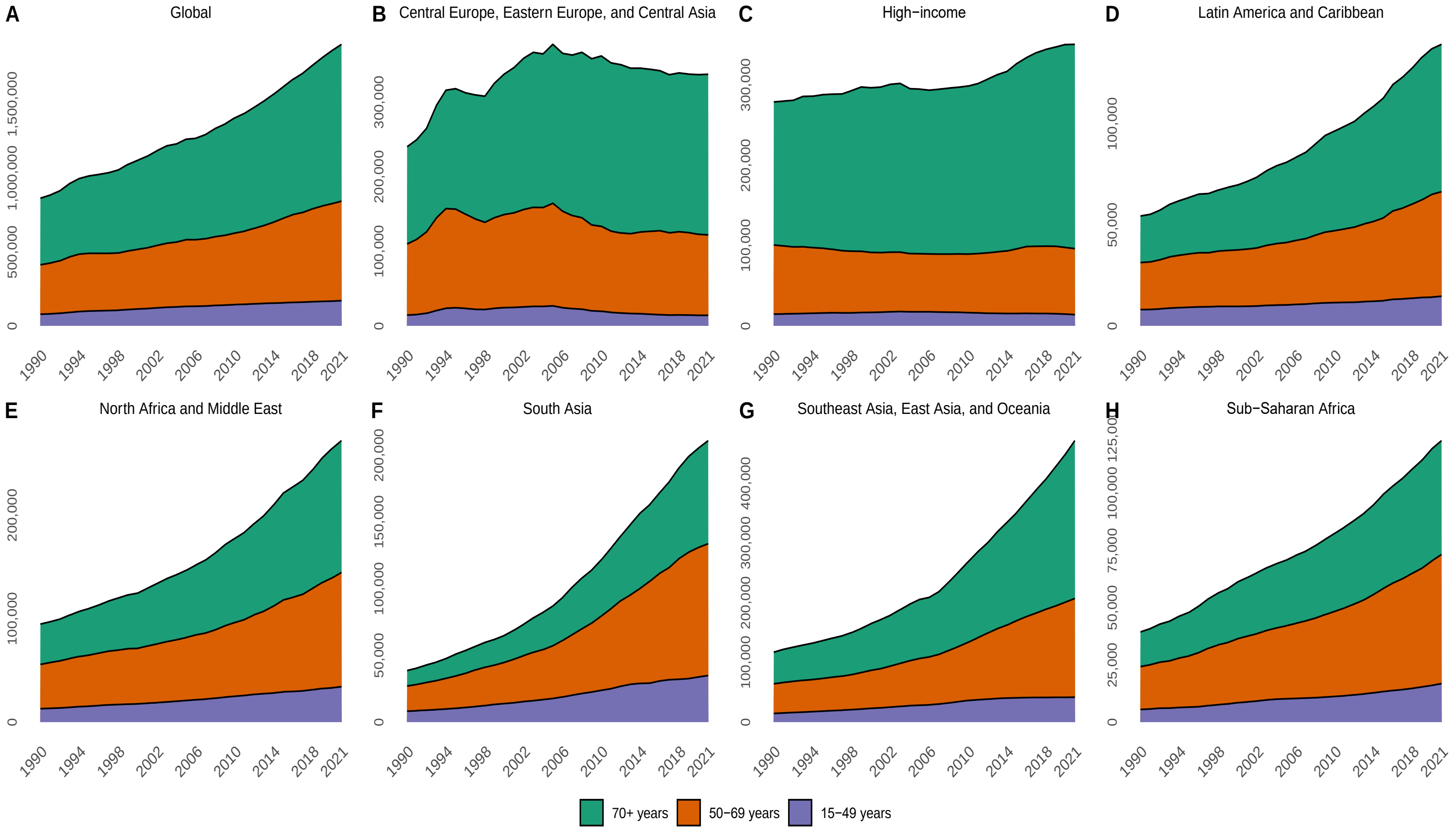

Supplementary Figure 3. Age-standardized Mortality Rate (ASMR) of Cardiovascular Disease Attributable to high Body Mass Index in Super-regions (1990–2021)

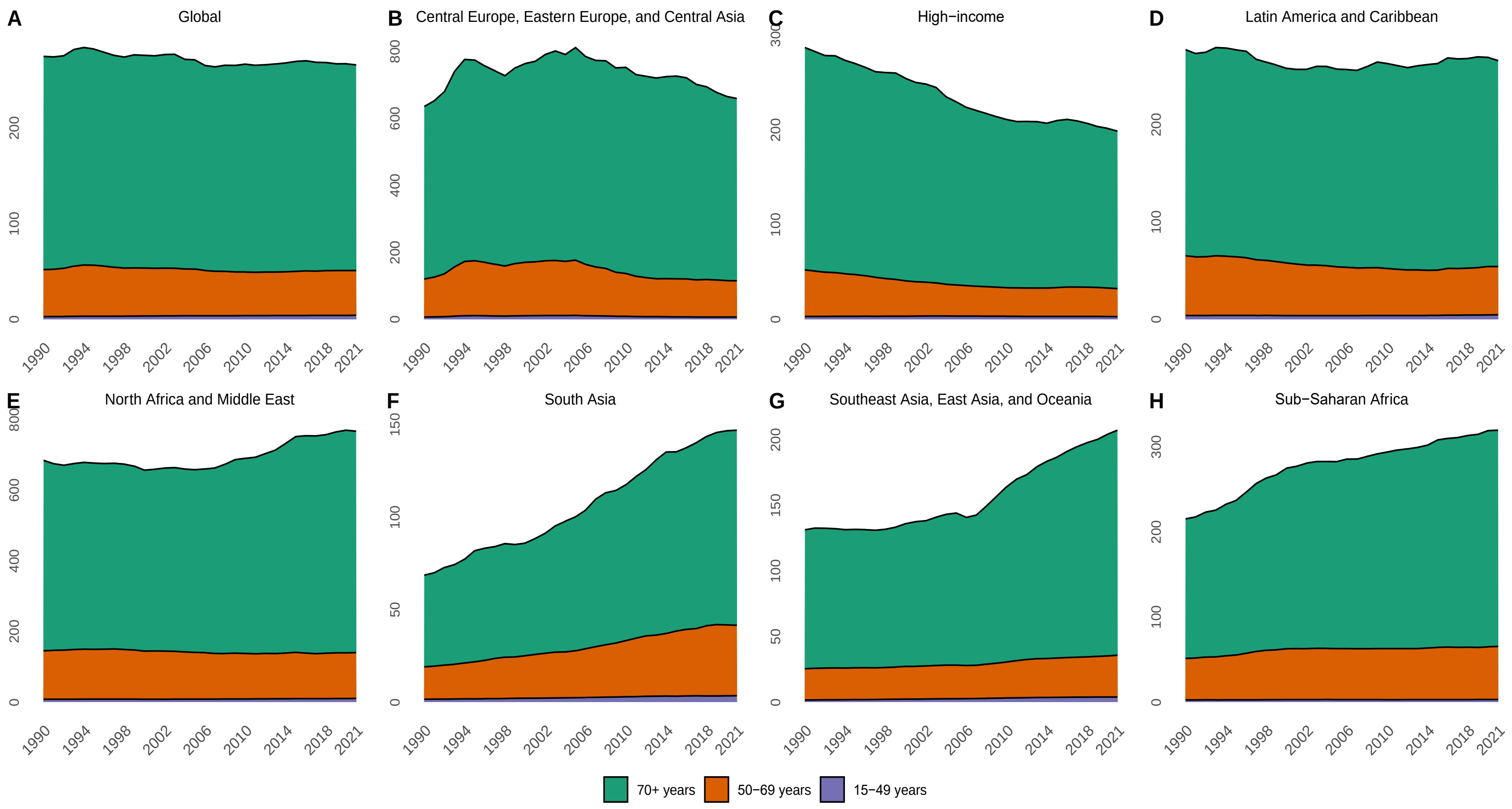

Supplementary Figure 4. Sensitivity Analysis of projected Cardiovascular Disease Mortality Attributable to High Body Mass Index (2022–2032) under varying Overdispersion Assumptions

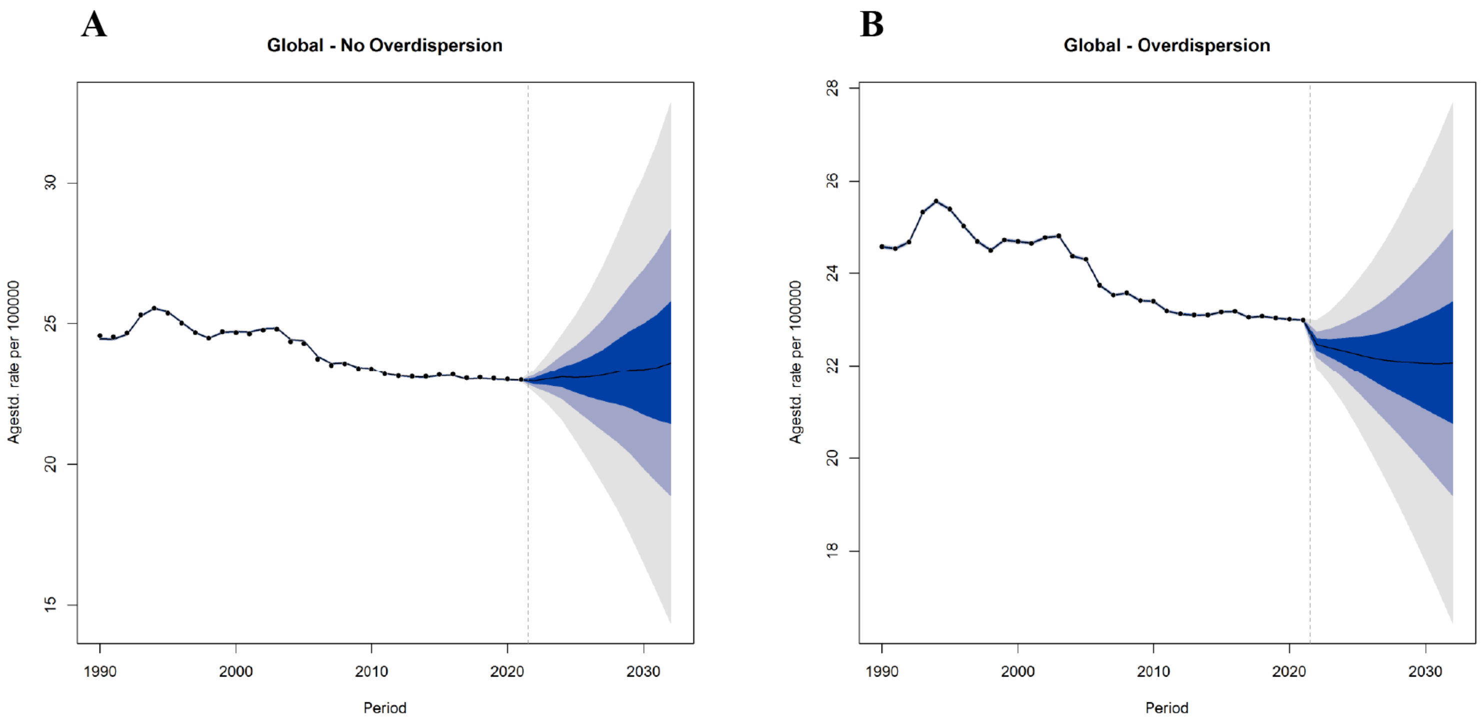

Supplementary Figure 5. Sensitivity Analysis of projected Cardiovascular Disease Mortality Attributable to High Body Mass Index (2022–2032) under varying Rate Assumptions

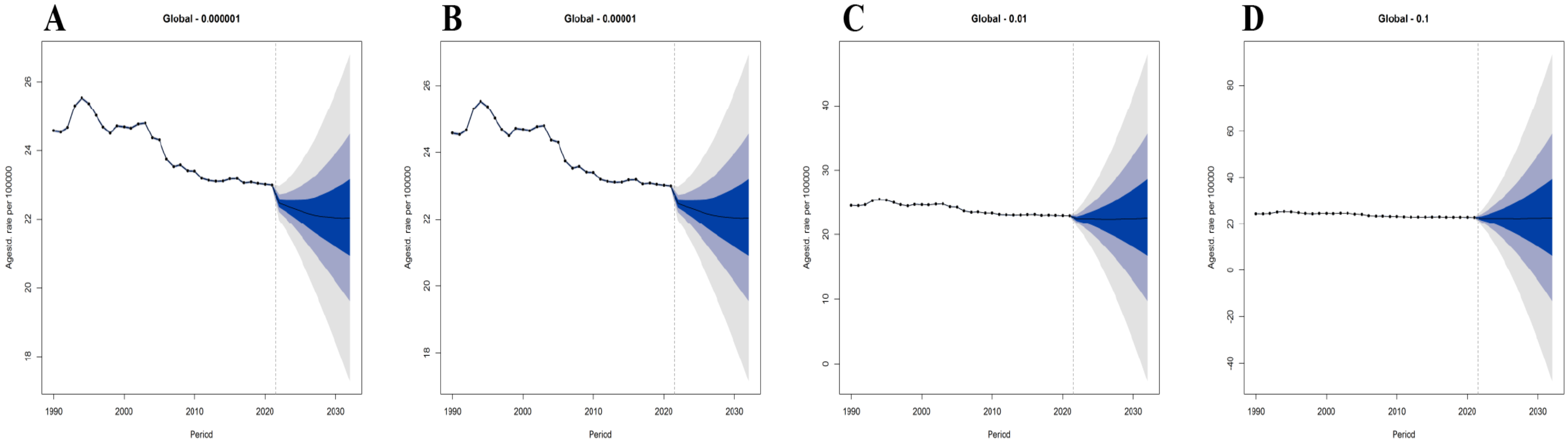

Supplementary Figure 6. Sensitivity Analysis of projected Cardiovascular Disease Mortality attributable to High Body Mass Index (2022–2032) under varying Shape Assumptions

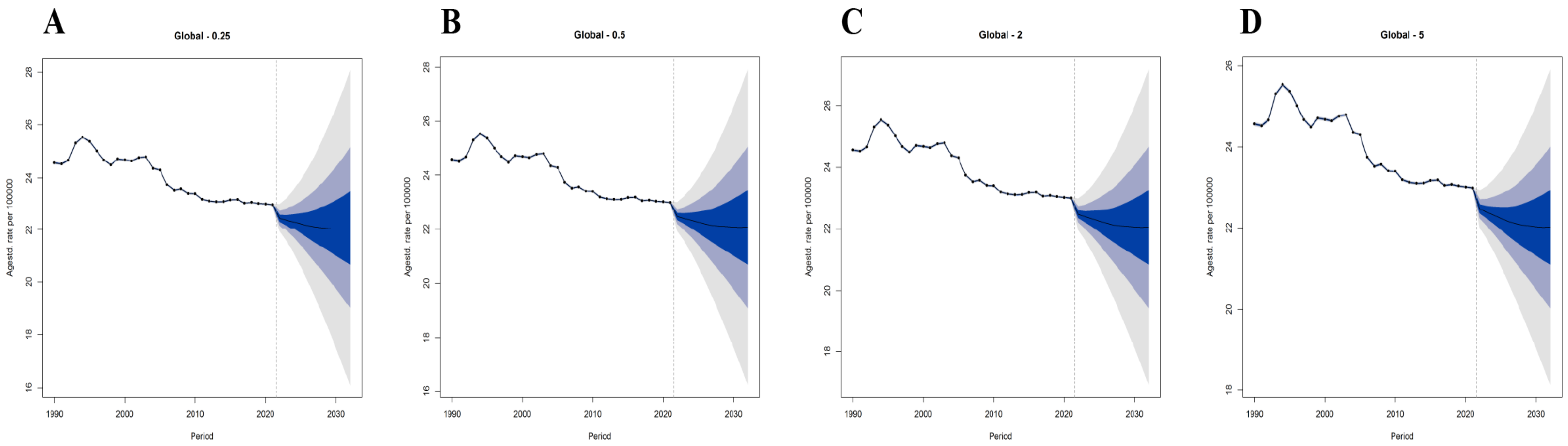

Supplement: Supplementary file 1 [file mmc1.pdf]
